# Hydrogen Sulfide: Recent Progression and Perspectives for the Treatment of Diabetic Nephropathy

**DOI:** 10.3390/molecules24152857

**Published:** 2019-08-06

**Authors:** Hai-Jian Sun, Zhi-Yuan Wu, Lei Cao, Meng-Yuan Zhu, Teng-Teng Liu, Lei Guo, Ye Lin, Xiao-Wei Nie, Jin-Song Bian

**Affiliations:** 1 Department of Pharmacology, Yong Loo Lin School of Medicine, National University of Singapore, Singapore 117597, Singapore; 2 School of Pharmaceutical Engineering and Life Science, Changzhou University, Changzhou 213164, China; 3 National University of Singapore (Suzhou) Research Institute, Suzhou 215000, China

**Keywords:** hydrogen sulfide, renal physiology, oxidative stress, renin-angiotensin system, diabetic nephropathy

## Abstract

Diabetic kidney disease develops in approximately 40% of diabetic patients and is a major cause of chronic kidney diseases (CKD) and end stage kidney disease (ESKD) worldwide. Hydrogen sulfide (H_2_S), the third gasotransmitter after nitric oxide (NO) and carbon monoxide (CO), is synthesized in nearly all organs, including the kidney. Though studies on H_2_S regulation of renal physiology and pathophysiology are still in its infancy, emerging evidence shows that H_2_S production by renal cells is reduced under disease states and H_2_S donors ameliorate kidney injury. Specifically, aberrant H_2_S level is implicated in various renal pathological conditions including diabetic nephropathy. This review presents the roles of H_2_S in diabetic renal disease and the underlying mechanisms for the protective effects of H_2_S against diabetic renal damage. H_2_S may serve as fundamental strategies to treat diabetic kidney disease. These H_2_S treatment modalities include precursors for H_2_S synthesis, H_2_S donors, and natural plant-derived compounds. Despite accumulating evidence from experimental studies suggests the potential role of the H_2_S signaling pathway in the treatment of diabetic nephropathy, these results need further clinical translation. Expanding understanding of H_2_S in the kidney may be vital to translate H_2_S to be a novel therapy for diabetic renal disease.

## 1. Introduction

Diabetic kidney injury is a leading cause of end-stage renal failure, predominantly due to the increase of diabetes and obesity [1,2]. Diabetic kidney disease is manifested by glomerular hypertrophy, deposition of extracellular matrix (ECM) proteins, expansion of mesangial matrix and glomerular basement membrane, tubulointerstitial fibrosis, excessive urinary loss of proteins, and loss of waste clearance function over time [3]. In fact, most of cardiovascular disease mortality for diabetic patients is related to diabetic kidney disease [4].

Despite optimal management, diabetic kidney disease is still a major contributor to morbidity and mortality of diabetic patients worldwide [5]. Accumulating evidence indicates that hyperglycemia mediates renal damage in diabetes via multiple molecular mechanisms including oxidative stress, proinflammatory cytokines, induction of transforming growth factor beta-1 (TGF-β1) expression, fibroblast and renin angiotensin system (RAS) activation, as well as depletion of adenosine triphosphate [6]. Though the great efforts to further understand the pathological mechanisms of diabetic kidney disease are continuous, the current therapies only retard the disease progression but cannot cure it. Thereby, there is a pressing demand to identify novel therapies or targets for the treatment of diabetic kidney disease.

Hydrogen sulfide (H_2_S) has been identified to exert a wide coverage of biological functions similar to other gasotransmitters nitric oxide (NO) and carbon monoxide (CO) [7]. Biochemical studies have demonstrated that kidneys are a rich source for H_2_S formation [3]. It is currently believed that cystathionine γ-lyase (CSE), cystathionine β-synthase (CBS) and 3-mercaptopyruvate sulfurtransferase (3-MST) are the dominated enzymes for H_2_S production in the kidney [8]. As reviewed recently, the potential roles of H_2_S in the regulation of glomerular filtration rate (GFR), sodium absorption, renin release and oxygen sensing in kidney system have been described in details [8,9]. Recent investigations support that H_2_S generation from the renal cells is reduced in acute or chronic kidney disorders including diabetic nephropathy [8,10,11,12]. In this review, we will discuss recent progression on H_2_S in both kidney physiology and diabetic kidney disease. Besides, we will discuss the recent experimental findings on the molecular mechanisms underlying the therapeutic effects of H_2_S against diabetic kidney disease and its possibilities, challenges for clinical application in the future.

## 2. Pathophysiology of Diabetic Kidney Disease

Diabetic kidney disease exhibits destructive structural changes such as glomerular basement membrane expansion, loss of podocytes, thickening of mesangial matrix and fusion of foot processes [13].

**Figure 1:**
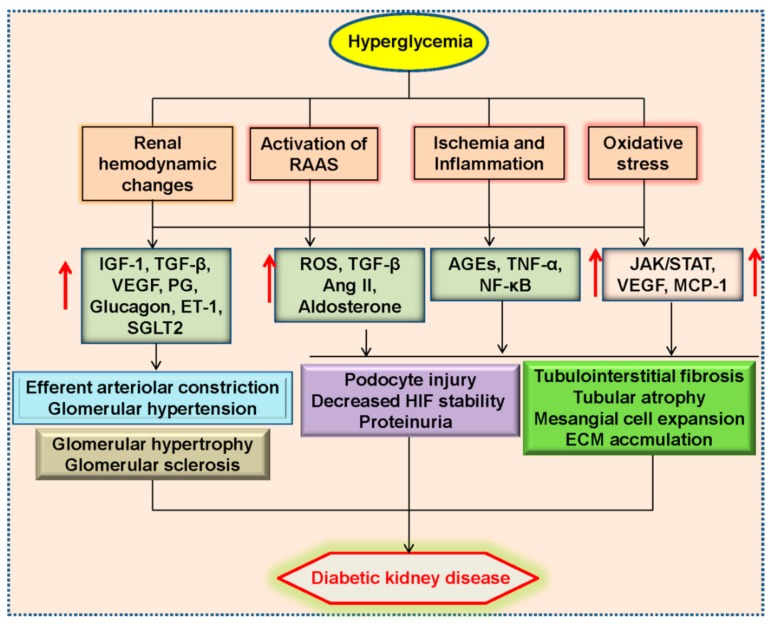
Conventional pathophysiology of diabetes kidney disease. Diabetic kidney disease is closely associated with renal hemodynamic changes, ischemia and glucose metabolism abnormalities, oxidative stress, inflammatory response and over-activated RAAS, which contributes to glomerular hypertension and sclerosis, tubulointerstitial fibrosis, tubular atrophy and mesangial cell expansion. RAAS, renin-angiotensin-aldosterone system; IGF-1, insulin-like growth factor 1; TGF-β1, transforming growth factor β1; VEGF, vascular endothelial growth factor; PG, prostaglandin; Ang II, angiotensin II; ET-1, endothelin-1; SGLT2, sodium glucose co-transporters 2; ROS, reactive oxygen species; AGEs, advanced glycation end products; TNF-α, tumor necrosis factor α; NF-κB, nuclear factor kappa-light-chain-enhancer of activated B cells; HIF, hypoxia-inducible factor; ECM, extracellular matrix; JAK/STAT, Janus kinase-signal transducer and activator transcription factor; MCP-1, monocyte chemotactic protein 1.

Conventionally, it is accepted that renal hemodynamics changes, oxidative stress, inflammatory response, hypoxia and renin-angiotensin-aldosterone system (RAAS) are majorly responsible for the pathogenesis of diabetic kidney disease [14] (Figure 1).

Glomerular hyperfiltration participates in the occurrence of diabetic kidney disease, and increased local angiotensin II (Ang II) induces the constriction of efferent arteriole, thus causing changes of autoregulation and glomerular hypertension [15]. At the same time, hyperglycemia and compensatory hyperinsulinemia promote vascular endothelial dysfunction through reactive oxygen species (ROS) production, activation of protein kinase C (PKC) and advanced glycation end-products (AGEs)-mediated proinflammatory response [16]. Activation of endothelin cells is necessary for podocyte injury and renal fibrosis in the diabetic kidneys [17].

Glomerular and renovascular lesions in diabetic kidney disease reduce the oxygen supply, thus eliciting renal medulla hypoxia and tubular dysfunction [18]. Without adequate oxygen supply, hyperglycemia may impair the stability of hypoxia-inducible factor (HIF) and facilitate renal tissue fibrosis [19]. Activation of RAAS is observed in renal cells induced by hyperglycemia and AGEs, this may be mediated by ROS and G protein-coupled metabolic receptor 91 (GPR91) [20,21]. Studies in animal models and clinical trials demonstrate that RAAS inhibition effectively slows the progression of diabetic kidney disease [22]. As an important member of RAAS, Ang II is critically involved in the process of renal tissue fibrosis and tubule dysfunction [23]. Aldosterone is also a key player in the pathophysiology of diabetic kidney disease via boosting macrophage infiltration and renal fibrosis [24].

Cellular and molecular experiments have shown that disturbed mitochondria function and endoplasmic reticulum stress, as well as abnormal activation of intracellular signaling pathways including nuclear factor kappa-light-chain-enhancer of activated B cells (NF-κB), Janus kinase-signal transducer and activator of transcription (JAK-STAT) pathway, have found to be integrally associated with diabetic kidney disease [14,25,26]. With further research, the novel fields of pathophysiology of diabetic kidney disease are gradually identified, such as genetic and epigenetic regulation as well as podocyte autophagy [14]. The completion of the molecular mechanism studies in these directions would improve our understanding of diabetic kidney disease, and also help us better screen treatment strategies for preventing the renal destruction associated with diabetes.

## 3. Expression of H_2_S in Diabetic Kidney Disease

H_2_S is a colorless gas with a rotten egg smell at room temperature and ambient pressure [27]. Despite its toxic effects as an environmental hazard, recent studies have highlighted its modulatory roles in fundamental cellular processes in several tissues. Distinct from the other gaseous transmitters like NO and CO, H_2_S is able to dissolve in water because of its weak acid property [28]. Analysis of physical and chemical properties has discovered that H_2_S can freely penetrate into cell membrane of all cell types due to it is highly lipophilic [29], thereby allowing H_2_S to exhibit a wide spectrum of phenomena including development, angiogenesis and carcinogenesis [7].

**Figure 2:**
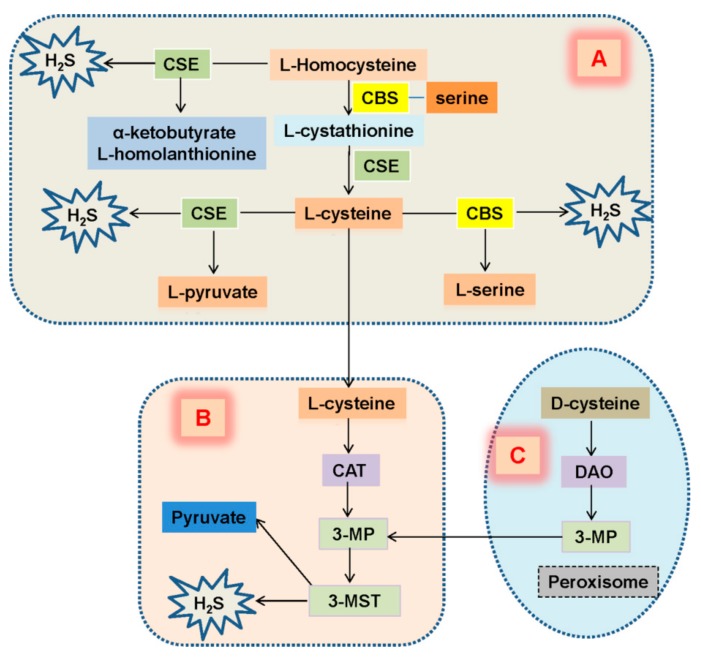
Endogenous synthesis of H_2_S in renal system. (**A**) CSE reacts with L-homocysteine to induce H_2_S generation accompanied by formation of α-ketobutyrate and L-homolanthionine. CBS catalyzes L-homocysteine that leads to the production of L-cystathionine, which is converted into L-cysteine by CSE. The presence of L-cysteine serves as a substrate for generation of H_2_S by CBS and CSE. (**B**) L-Cysteine translocates to mitochondria, followed by conversion to 3-MP by CAT. 3-MST produces H_2_S generation from 3-MP. (**C**) Peroxisome-mediated generation of 3-MP from D-cysteine with the aid of DAO. 3-MP is then imported into the mitochondria and becomes a substrate for 3-MST to generate H_2_S. CBS, cystathionine β-synthase; CSE, cystathionine g-lyase; 3-MST, 3-mercaptopyruvatesulfurtransferase; CAT, cysteine aminotransferase; DAO, D-amino acidoxidase; 3-mercapto pyruvate, 3-MP.

In mammalian cells, H_2_S is primarily generated by the enzymes including CBS, and CSE in the cytosol through the trans-sulfuration pathway [30,31,32]. Another enzyme, 3-MST gives rise to the endogenous H_2_S production in the mitochondria using 3-mercapto pyruvate (3-MP) as a substrate [33,34]. Additionally, homocysteine is condensed with serine by CBS to generate cystathionine, which is converted into l-cysteine by CSE. The presence of l-cysteine is served as a substrate for both CBS and CSE to produce H_2_S. CSE also produces H_2_S by catalyzing homocysteine into homolanthionine [30]. As reviewed recently, two main pathways are proposed to be involved in H_2_S generation in the kidney, such as the trans-sulfuration pathway and the D-cysteine pathway [3,35]. In the kidney, D-cysteine may generate more H_2_S relative to the L-cysteine pathway [36]. The mechanisms underlying the H_2_S-generated pathways in the kidney are summarized in Figure 2.

Similar to liver and lung, CSE, CBS and 3-MST are highly expressed in the kidney [37]. CBS is predominantly located in renal proximal tubules, while CSE is mainly expressed in renal glomeruli, proximal tubules, interstitium, and interlobular arteries [38,39]. Specifically, immunohistochemistry results have shown that CSE is present in glomerular endothelial and mesangial cells, podocytes, proximal and distal tubules as well as the peritubular capillaries and blood vessels [40]. Besides, 3-MST can be detected in proximal tubular epithelium in the kidney [37]. CBS and CSE are abundantly expressed in renal tissues that synergistically produce H_2_S in the kidney [41]. Under normal conditions, the expression of CSE protein in the kidney is 20-fold higher than CBS, thus, CSE may be the main H_2_S-forming enzyme in the kidneys [42,43].

Decrease in endogenous H_2_S is reported to participate in the pathogenesis of diabetic nephropathy [41,44]. Treatment with sodium hydrosulfide (NaHS, a H_2_S donor) ameliorates kidney lesions in type 2 diabetes via increasing glucose uptake in both myotubes and adipocytes [44]. Suppressed CSE-catalyzed endogenous H_2_S production by hyperglycemia may play an important role in the pathogenesis of diabetic nephropathy [45]. In type 2 patients, the plasma H_2_S level is lower than that in normoglycemic humans [46], and it is negatively correlated with the markers of adiposity [47]. Moreover, the diabetic patients with dialysis exhibit reduced level of plasma H_2_S, and lower H_2_S level may contribute to uremic atherosclerosis in chronic hemodialysis patients with diabetic nephropathy [48]. Recent documents have demonstrated that renal H_2_S-producing enzymes CBS and CSE are downregulated in animals with type 1 or type 2 diabetes [45,49,50]. The above evidence indicates that H_2_S deficiency contributes to the development of diabetic kidney injury.

## 4. H_2_S Regulation of Renal Function

### 4.1. H_2_S and Renal Excretory Function

It has been reviewed that H_2_S participates in the regulation of renal functions [41]. In anesthetized Sprague-Dawley rats, intrarenal arterial infusion of H_2_S donor NaHS results in increased renal blood flow, GFR, urinary sodium (U(Na) × V), and potassium (U(K) × V) excretion, this effect is mimicked by renal artery infusion of L-cysteine, a H_2_S generating substrate [41], suggesting that H_2_S plays a tonic role in the regulation of renal function under physiological condition. In that study, the authors propose that H_2_S augments urinary excretion of sodium and potassium by the inhibition of Na^+^-K^+^-2Cl^−^ cotransporter and Na^+^-K^+^-ATPase [41]. Mechanistic studies have shown that H_2_S directly induces endocytosis and inhibition of Na^+^-K^+^-ATPase via phosphatidyl inositol 3 kinase (PI3K)/protein kinase B (Akt) pathway in renal tubular epithelial cells [51]. In vitro studies have identified that H_2_S inhibits Na^+^-H^+^ exchanger-3 activity in tubular epithelial cells [51]. It may be important to examine whether H_2_S directly affects the expressions of these transporters.

Patch clamp studies indicate that hydrogen peroxide augments PI3K activity to activate the epithelial sodium channel (ENaC), this is corrected by H_2_S [52]. It is also demonstrated that H_2_S prevents β-adrenergic agonist-induced ENaC-mediated Na transport in human lung epithelial cells [53]. The Cl^−^/HCO_3_^−^ exchanger activity in aortic tissues and vascular smooth muscle cells is dampened by H_2_S [54,55]. Unfortunately, the effect of H_2_S on ENaC and Cl^−^/HCO_3_^−^ exchanger activities in renal system is still undefined. Considering the indispensable role of ENaC and Cl^−^/HCO_3_- exchanger in the control of sodium reabsorption and homeostatic maintenance of physiological pH, it will be interesting to understand the action of H_2_S on ENaC and Cl^−^/HCO_3_^−^ exchanger activities as well as subsequent renal function.

### 4.2. H_2_S and Oxygen Sensing

Despite the production of H_2_S is independent of oxygen, the metabolism of H_2_S is largely linked with the presence of oxygen [56]. The role of H_2_S in oxygen sensing has been tested in the carotid body, which participates in ventilation, heart rate and blood pressure [57]. In addition, emerging physiological evidence for H_2_S-mediated oxygen sensing has been clarified in cardiovascular system [58,59], respiratory system [60] and gastrointestinal tract [61]. In the kidney, partial pressure of oxygen declines from cortex to medulla in normal rats [62]. The recognition of H_2_S as an oxygen sensor in the kidney, especially in medulla, has been identified [11]. Compared with the renal cortex, the oxygen content is lower in medullar, resulting in a higher level of H_2_S in this area [11]. In the mitochondria, H_2_S is regarded as an electron donor for adenosine triphosphate release [63,64]. It is still unclear whether H_2_S can be a possible source of energy in renal medulla. At least, reduction of oxygen leads to H_2_S generation, which is helpful to recovery oxygen supply through raising medullary blood flow and suppressing tubular transport [65]. However, the downstream effectors of H_2_S-mediated oxygen sensor in renal system remain to be further elucidated. Notably, the excessive erythropoietin levels are synthesized in anemia and hypoxia kidney [66,67]. Given H_2_S participates in oxygen sensing, whether H_2_S exerts a regulatory role in erythropoietin synthesis in the kidney, needs in depth exploration.

## 5. Role of H_2_S in Diabetic Kidney Disease

### 5.1. Renin–Angiotensin System (RAS) and H_2_S in Diabetic Nephropathy

The RAS is a multistep enzymatic cascade that plays a role in the control of blood pressure and fluid homeostasis [68]. Angiotensinogen is a major substrate of this cascade, and the 10 amino acids from the N-terminus of angiotensinogen are specifically cleaved to form angiotensin I. The conversion of angiotensin I to Ang II by angiotensin-converting enzyme (ACE) is a central step in the classical RAS pathway [22]. The initial evidence for a role of the RAS in diabetic nephropathy is established in a rat model of diabetes induced by streptozotocin (STZ) [69]. It is suggested that the dysregulated glomerular hemodynamic profile in diabetic rats might be associated with activation of the RAS [70,71]. Further studies unveil that hyperglycemia stimulates the renal RAS, and renders an increase in glomerular hydrostatic pressure, proteinuria, and structural injury with sclerosis and fibrosis [22]. Blockade of the RAS has been shown to delay the progression of diabetic kidney disease [72]. Over-activation of intrarenal RAS aggravates diabetic nephropathy due to a fact that inhibition of RAS retards the progression of diabetic nephropathy in animal models and clinical trials [22,72]. It is likely that intrarenal RAS is activated in patients suffering from diabetes, thereby RAS blockers have the benefits for abnormal glomerular structures in patients with diabetic kidney disease [73,74,75]. Present researches recommend that RAS blockers can relieve the nephropathy for albuminuric patients with diabetes [14]. These above studies would offer great promise for RAS-inhibition-based therapies in diabetic nephropathy.

Renin is mainly produced from the so-called juxtaglomerular epithelioid cells, and this process is regulated by intracellular cyclic adenosine monophosphate (cAMP) level [76]. Actually, H_2_S is capable of downregulating the intracellular cAMP level in various cell types [77,78], implying that H_2_S may regulate renin release. It has been reported that H_2_S treatment inhibited the upregulation of renin level in renovascular hypertensive rats accompanying with a reduction of intracellular cAMP level in primary cultures of renin-rich kidney cells [78,79]. We also find that H_2_S inhibits forskolin-induced renin degranulation in mast cells by lowering intracellular cAMP level, thus protecting against isoproterenol (ISO)-induced heart failure [80]. Likewise, H_2_S therapy protects multiple organs including the heart, kidney, and blood-vessels and improves exercise capacity, coupled with inactivation of RAS in a murine model of transverse aortic constriction-induced heart failure [81]. These existing results show that H_2_S may modulate renal activity and release, which may be beneficial for the prevention of diabetic nephropathy. However, the researches regarding the direct effect of H_2_S on renin activity and release in diabetic kidney disease are still rare.

**Figure 3:**
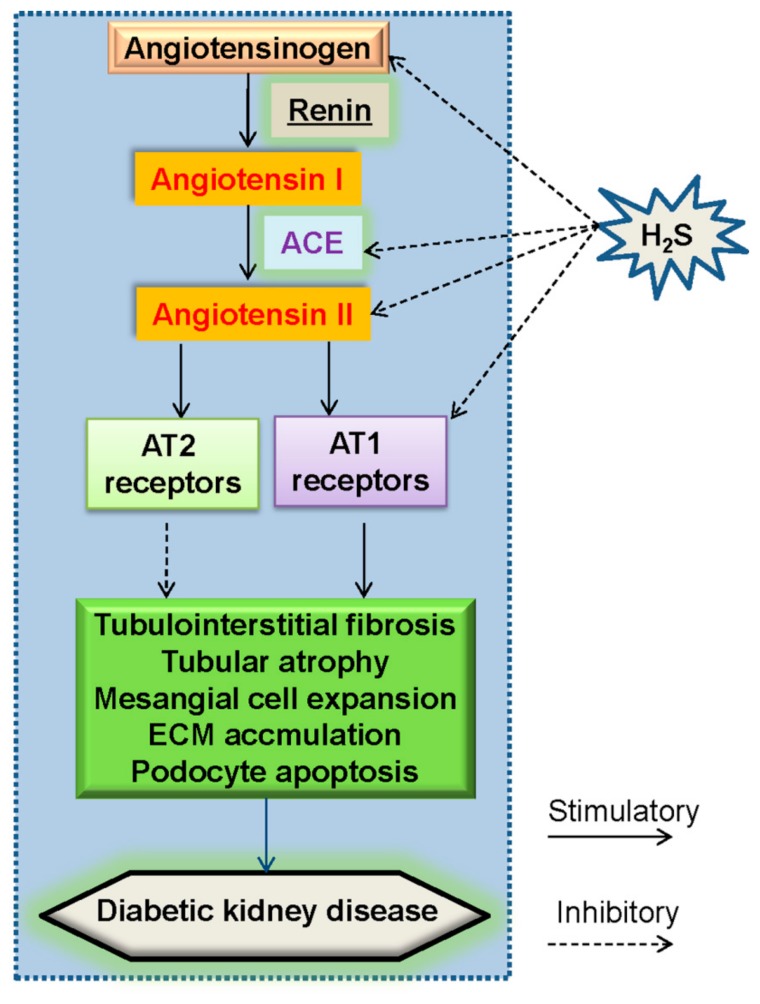
A proposed sketch showing the effect of H_2_S on the renin-angiotensin-system (RAS). Angiotensinogen is cleaved by renin to produce angiotensin I, then angiotensin I is further converted to Ang II by ACE. The deleterious effects of Ang II are mediated by AT1 receptors, whereas Ang II acts on AT2 receptors to function as a negative modulator of AT1 receptor actions. The activated RAS in diabetic kidney disease was ameliorated by H_2_S treatment via inhibition of angiotensinogen, ACE, Ang II and AT1 receptors. RAS, renin-angiotensin-system; ACE, angiotensin converting enzyme; Ang II, angiotensin II.

Mounting evidence suggests that hyperglycemia acts synergistically with Ang II to promote renal cellular injury in diabetes [82]. In proximal renal tubules, increased Ang II synthesis induces interstitial fibrosis [83]. The two main Ang II receptors (AT1 and AT2) have been cloned and well characterized [83]. Most biological effects of Ang II are mediated by the AT1 receptors [84]. Accordingly, it has been demonstrated that Ang II receptor blockers have a slowing effect on the progression of diabetic nephropathy [85]. Within RAS, high glucose stimulation upregulates mRNA levels of angiotensinogen (AGT), angiotensin converting enzyme (ACE), Ang II type I receptor (AT1) mRNA levels when compared with that of normal glucose-treated cells, which are reversed by supplementation of H_2_S [86]. The protein expressions of ACE and AT1 receptors as well as Ang II are significantly upregulated in the diabetic kidneys and downregulated after treatment with H_2_S donor, NaHS [50]. These data suggested H_2_S alleviated the development of diabetic nephropathy by partially attenuating RAS activity (Figure 3).

### 5.2. Oxidative Stress and H_2_S in Diabetic Nephropathy

A plethora of molecules are implicated for ROS generation, such as, reduced nicotinamide adenine dinucleotide phosphate (NADPH) oxidase, uncoupled nitric oxide synthase (NOS) and mitochondrial respiratory chain via oxidative phosphorylation [87,88]. Excessive accumulation of ROS activates PKC, MAPK, and numerous cytokines and transcription factors, which eventually lead to increased ECM production and progressive fibrosis in diabetic kidney disease [89]. High glucose induces substantial ROS generation in glomerular mesangial cells [90].

Antioxidants inhibit high glucose- and hydrogen peroxide-induced TGF-β1 and fibronectin upregulations in mesangial cells and tubular epithelial cells, thus providing evidence that ROS are necessary for high glucose-induced renal injury [89,91,92,93]. Redundant ROS trigger signal transduction cascade and transcription factors as well as their downstream molecules, which are implicated in glomerular mesangial expansion and tubulointerstitial fibrosis [89]. In the kidney, the excessive oxidative stress and its associated inflammation have been found to aggravate diabetic nephropathy in recent years [94]. These observations suggest that ROS may act as intracellular messengers in diabetic kidney disease, thus making it an attractive target for developing novel therapeutic strategies to ameliorate diabetic nephropathy.

**Figure 4:**
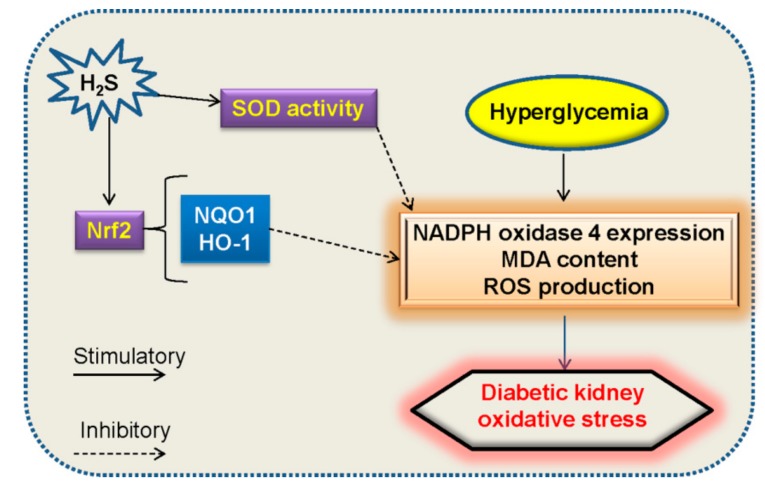
Effects of H_2_S on oxidative stress in diabetes kidney disease. H_2_S is found to reduce high glucose-induced oxidative stress by activating the Nrf2 antioxidant pathway and two downstream targets of Nrf2, HO-1 and NQO1, as well as enhancing the SOD and glutathione peroxidase activities in diabetes kidney disease. Nrf2, nuclear factor erythroid-2 related factor 2; HO-1, heme oxygenase-1; NQO1, NADPH: quinone oxidoreductase-1; SOD, superoxide dismutase.

Supplementation with H_2_S attenuates high glucose-induced elevation in ROS production in renal mesangial cells and diabetic rat kidneys [86]. H_2_S treatment protects the kidneys of type 1 diabetic rats, which may be related to the suppression of oxidative stress via incrementing the activities of superoxide dismutase (SOD) [95]. Exposure to high glucose promotes ROS generation in mesangial cells, and the effect is counteracted by NaHS [45]. High glucose increases NADPH oxidase 4 (NOX4) expression in renal proximal tubular epithelial cells, this change is inhibited by NaHS [49]. These above studies provide the direct evidence that H_2_S plays an important role in the renal cellular response to high glucose-induced oxidative stress (Figure 4). Thus, application of H_2_S donors seems to be a choice for the treatment of diabetic nephropathy.

Nuclear factor erythroid-2 related factor 2 (Nrf2) is a transcription factor with a basic leucine zipper motif [96]. This transcription factor is highly conserved with Nrf2-ECH homology domains, Neh1 to Neh7 [97,98]. Among which, Neh1 facilitates the nuclear translocation of Nrf2 [99], while Neh2 enables the coupling of Nrf2 with Kelch-like ECH-associated protein 1 (Keap1) [100,101]. In addition, the lysine residues and a serine residue within Neh2 domain are inhibitory for proteasome-mediated Nrf2 degradation [102]. The Neh3–Neh5 domains are necessary for Nrf2 transcriptional activity [103], and Neh6 domain is responsible for Nrf2 degradation [104]. With respect to Neh7 domain, it is identified to suppress Nrf2 downstream gene expressions via binding to the retinoic acid receptor α [105]. Under normal status, Keap1 functions as an endogenous inhibitor of Nrf2 by interacting with Nrf2 to form a complex [106,107]. During oxidative stress, the modification of three important cysteine residues of Keap1 leads to Nrf2 liberalization, followed by Nrf2 nuclear translocation [108,109]. After that, in the nucleus, Nrf2 promotes the induction of phase II detoxification enzymes and antioxidants [110]. The activity of Nrf2 plays a central role in cellular resistance to oxidative stress [111]. In fact, Nrf2-related signaling pathway is demonstrated to be crucial in maintaining the balance between oxidation and reduction in the kidney, thus activation of Nrf2 pathway appears to be an effective method for the treatment of diabetic kidney disease [112,113,114]. In regard this, it may be interesting to know whether H_2_S activates Nrf2 pathway to prevent oxidative stress in disease. As expected, H_2_S could activate the Nrf2 signaling pathway together with the upregulations of antioxidant proteins haem oxygenase-1 (HO-1) and NAD(P)H: quinone oxidoreductase 1 (NQO1), thus alleviating the development of diabetic cardiomyopathy via attenuation of oxidative stress in the heart [115]. Upon exposure to high glucose plus oxidized low density lipoprotein (ox-LDL), the superoxide anions and adhesion molecule levels are elevated in endothelial cells [116]. However, administration of H_2_S rectifies these dysregulated changes via increasing S-sulfhydration of Keap1, interfering with the interaction between Keap1 and Nrf2, as well as stimulating Nrf2 nuclear translocation [116]. S-propargyl-cysteine, a novel molecule that upregulates endogenous H_2_S production, is reported to attenuate the generation of ROS and apoptosis in diabetic cardiomyocytes, this effect may be dependent on increased stability and nuclear translocation of Nrf2 and the dissociation of Nrf2 from Keap1 [117]. In cisplatin-induced nephrotoxicity, we demonstrate that hydrogen polysulfide, a novel H_2_S-derived signaling molecule, leads to the nucleus translocation of Nrf2 in renal proximal tubular cells [118]. As a result, hydrogen polysulfide mitigates cisplatin-induced renal cell oxidative stress and apoptosis [118]. Similar to these observations, H_2_S is found to activate the Nrf2 antioxidant pathways, thereby decreasing malondialdehyde (MDA) levels and restoring SOD and glutathione peroxidase activities in diabetic kidney [50]. To be noticed, further studies are required to elucidate the precise behavior of H_2_S-mediated suppression of oxidative stress via Nrf2 pathway in diabetic kidney disease. Anyway, activation of Nrf2 pathway by H_2_S might serve as a promising strategy for the treatment of oxidative stress-associated kidney damage under diabetic condition.

### 5.3. Inflammation and H_2_S in Diabetic Kidney Disease

The intimate mechanisms contributing to the progression of diabetic renal injury are not well elucidated, but current knowledge indicates that immune and inflammation responses appear to be relevant factors [119]. Growing evidence suggests that pathogenesis of diabetes mellitus is widely linked with the innate immune system activation and a chronic low-grade inflammatory state [120,121]. It has been accepted that pro-inflammatory signaling pathways and their downstream effectors are emerging as promising therapeutic targets for diabetic nephropathy [122]. In the kidney, many intrinsic renal cells including glomerular, endothelial, tubular, and mesangial cells are capable of synthesizing inflammatory cytokines, and these substances are gradually upregulated as diabetic nephropathy progresses, suggesting a role of inflammation in the pathogenesis of diabetic kidney disease [123].

In the present time, inflammatory cytokines activate diverse transduction pathways in the pathogenesis of diabetic kidney, such as oxidative stress and transcription factors including NF-κB and JAK/STAT pathways [124,125]. In addition, macrophages and T lymphocytes within kidneys also play determinant roles in diabetic renal damage, and their accumulation is positively related with the severity of diabetic nephropathy experimental models [1]. Certainly, inflammation response is an important contributor to the pathogenesis of diabetic nephropathy, this participation involves increased chemokine and pro-inflammatory cytokine production, infiltration of inflammatory cells to the kidney, and subsequent renal damage. Such in-depth exploration of inflammatory response may identify novel anti-inflammatory approaches for the treatment of diabetic nephropathy.

Recently, it is widely accepted that H_2_S inhibits inflammatory cytokine production, and suppresses activation of key transcriptional factors [126]. In renal system, administration of H_2_S donors ameliorates renal ischemia/reperfusion injury accompanied by reductions in oxidative stress and inflammatory response [3]. In unilateral obstruction kidney injury, the renal H_2_S generation is declined, and application of NaHS obviously retards the kidney injury, including inflammatory response [38]. It can be concluded that H_2_S metabolism is dysregulated in inflammatory diseases including diabetic nephropathy.

**Figure 5:**
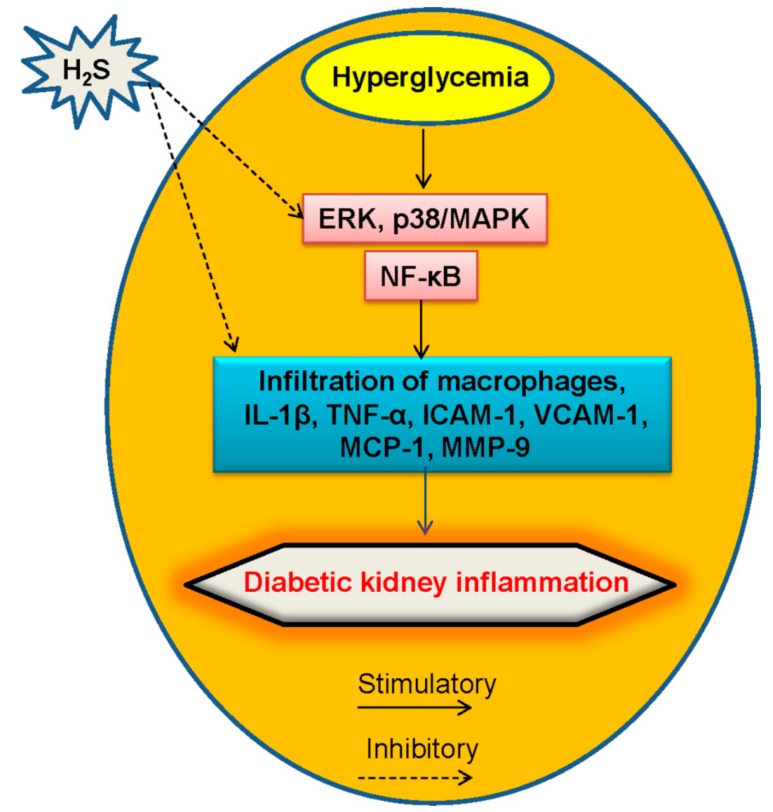
A proposed model of diabetic kidney inflammation mediated by H_2_S. The anti-inflammation mechanisms of H_2_S may involve its inhibition of macrophages infiltration, as well as its blockade of NF-κB and MAPK signaling in renal system. NF-κB, nuclear factor kappa-light-chain-enhancer of activated B cells; MAPK, mitogen-activated protein kinase, TNF-α, tumor necrosis factor α; IL-1β, interleukin-1β; VCAM-1, vascular cell adhesion molecule-1; ICAM-1, intercellular adhesion molecule-1; MCP-1, monocyte chemotactic protein-1; MMP-9, matrix metalloproteinase-9.

In STZ-induced diabetic rats, H_2_S donor NaHS exerts an-inflammatory actions through inhibiting NF-κB signaling in rat glomerular mesangial cells [50], thus alleviating the development of diabetic nephropathy. MMP-9 is a zinc-dependent endopeptidase, which can be activated by ROS [127]. As an inflammatory cytokine, inflammatory cell-derived MMP-9 leads to ECM degradation and renal vascular remodeling [128,129]. Hyperglycemic Akita mice exhibit higher level of MMP-9 and lower production of H_2_S, and H_2_S treatment reverses the altered diabetic renal remodeling induced by MMP-9 [130]. Therefore, H_2_S has therapeutic potential to ameliorate diabetic renal remodeling in association with suppression of inflammation response (Figure 5). However, the regulation of H_2_S in the diabetic kidney inflammation still warrants more investigation.

## 6. Renal Fibrosis and H_2_S in Diabetic Kidney Disease

Renal fibrogenesis is a complex process involving a host of pathological scarring processes such as dysregulated ECM assembly, anchoring or degradation in glomerular basement membranes and the tubulointerstitium, as well as activated fibroblasts [131,132,133]. Moreover, epithelial mesenchymal transition (EMT) and endothelial mesenchymal transition (EndMT) programs are also considered as alternative mechanisms for renal fibrosis [134]. EndMT is responsible for the deposition of activated fibroblasts and myofibroblasts in diabetic kidney fibrosis [135]. It is well known that even after control of blood glucose level, diabetic patients may continue to develop to glomerular and tubulointerstitial fibrosis, eventually resulting in renal failure [136]. These observations suggest that renal fibrosis may play a significant role in the pathobiology of diabetic kidney disease.

### 6.1. EndMT and H_2_S in Diabetic Renal Fibrosis

In such fibrotic process, renal fibroblasts play prominent roles in the development of diabetic renal fibrosis [137]. Activated fibroblasts may be from resident quiescent fibroblasts via the process of EndMT [135]. It has been shown that activated fibroblasts are correlated with the excess deposition of interstitial ECM in diabetic kidney disease [138]. The emergence of EndMT is responsible for the accumulation of activated fibroblasts in renal fibrosis, suggesting that targeting EndMT might have therapeutic potential for diabetic renal fibrosis.

Vascular endothelial cells may be transformed to fibroblasts by undergoing a phenotypic transition similar to EMT, referred to as EndMT [139]. The process of EndMT involves the upregulated expressions of mesenchymal proteins including α-smooth muscle actin (α-SMA) and the loss of endothelial markers including endothelial cadherin (E-cadherin) and CD31 [140]. The vital role of EndMT in renal fibrosis is recently discussed and reviewed [23,141]. The first evidence for the EndMT in renal fibrosis is established by Zeisberg and colleagues, their results demonstrate that a large proportion of myofibroblasts coexpress the endothelium marker CD31 in three mouse models, unilateral ureteral obstruction (UUO), genetic modification, STZ-induced diabetic nephropathy [135], suggesting that these fibroblasts are likely derived from endothelial cells and EndMT may substantially contribute to the development and progression of renal fibrosis. Another group also confirms that EndMT occurs and leads to the formation of myofibroblasts in early diabetic renal fibrosis [142]. Interestingly, under endoplasmic reticulum stress, H_2_S blocks the EndMT process in human umbilical vein endothelial cells (HUVECs) via downregulating the mesenchymal marker expressions, and upregulating the endothelial marker expressions [143]. In cultured kidney fibroblasts, H_2_S inhibited the excessive cell proliferation. Furthermore, the differentiation of renal fibroblasts to myofibroblasts is antagonized by H_2_S donor, which is associated with inhibition of TGF-β1-Smad and MAPK signaling pathways [38]. However, no studies are conducted to determine whether H_2_S directly modulates the EndMT process in diabetic renal fibrosis.

### 6.2. EMT and H_2_S in Diabetic Renal Fibrosis

EMT is a pathological process in which epithelial cells lose epithelial characteristics and acquire properties of mesenchymal cells [131]. EMT has been divided into three subtypes on the basis of their functional consequences and biomarker context [144]. Type 1 EMT is a key modulator in the formation of diverse cell types without organ fibrosis, whereas type 2 EMT is related with the transition of epithelial cells to tissue fibroblasts in the process of organ fibrosis [144]. Meanwhile, type 2 EMT would continue in response to inflammation, resulting in persistent organ fibrosis and tissue destruction [145]. Type 3 EMT is detected in carcinoma cells, this process is a prominent contributor to tumor invasion, migration, and metastatic outgrowth [146]. It is highlighted that type 2 EMT is a direct contributor to the generation of myofibroblast population in the kidney, thus inducing the development of diabetic renal fibrosis [147]. In general, it is recognized that the transformation of tubular epithelial cells into mesenchymal cells is the most possible mechanism that underlies diabetic kidney fibrosis [148]. The existence of EMT could be initiated by a myriad of molecules, in which TGF-β1 seems to be a primary player [149,150,151].

Following UUO male Lewis rats, the uncontrollable expressions of EMT markers are mitigated upon H_2_S treatment [152]. Daily treatment with the slow-releasing H_2_S donor GYY4137 attenuates renal injury by regulating the TGF-β1-mediated EMT pathway in UUO model [153,154]. Consistently, H_2_S counteracts Ang II- and TGF-β1-induced EMT via inactive TGF-β1 monomer formation in renal tubular epithelial cells [155]. Under diabetic condition, H_2_S levels in the plasma and renal cortex are evidently reduced, while the levels of TGF-β1 and collagen IV are enhanced in STZ-induced diabetic rats, which are reversed by administration of NaHS [45]. H_2_S inhibits TGF-β1-induced EMT process in renal tubular epithelial cells, as evidenced by downregulated expressions of α-SMA and fibronectin, and upregulated expression of E-cadherin, and blockade of ERK- and β-catenin-dependent pathways may account for the protective effect of H_2_S [156]. In the process of EMT, future studies elucidating other targeted molecules induced by H_2_S in diabetic renal fibrosis will allow a better understanding of comprehensive cellular responses to H_2_S.

**Figure 6:**
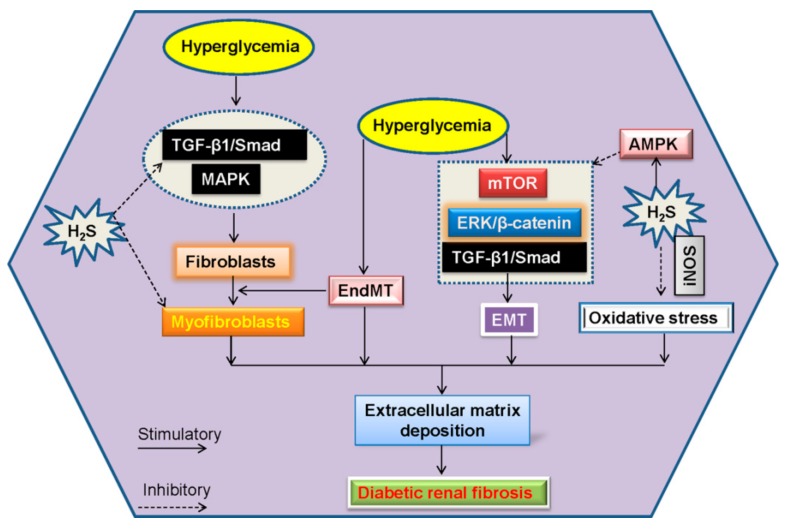
Effect of H_2_S on renal fibrosis in diabetic kidneys. Activated fibroblasts may be from resident quiescent fibroblasts via the process of EndMT. H_2_S treatment prevents the differentiation of quiescent renal fibroblasts to myofibroblasts and myofibroblasts proliferation via inhibition of the TGF-β1/Smad and MAPK signaling pathways. Blockade of ERK- and β-catenin-dependent pathways may be involved in the protective effect of H_2_S on the formation of EMT in renal tubular epithelial cells. In addition, H_2_S dose-dependently stimulates AMPK phosphorylation and induces its subsequent inhibition of mTORC1 activity. Induction of iNOS, is required for H_2_S to inhibit high glucose-induced oxidative stress and matrix protein generation in renal proximal tubular epithelial cells. EndMT, endothelial-mesenchymal transition; EMT, epithelial mesenchymal transition; TGF-β1, transforming growth factor-β1; MAPK, mitogen-activated protein kinase; mTORC1, mammalian target of rapamycin complex 1; ERK, extracellular regulated protein kinases; AMPK, adenosine 5’-monophosphate (AMP)-activated protein kinase; iNOS, inducible nitric oxide synthase.

Upon high glucose challenge, the mTORC1 signaling is activated due to the inhibition of AMPK activity in renal epithelial cells, thus stimulating matrix protein synthesis and renal hypertrophy [157]. NaHS dose-dependently stimulates AMPK phosphorylation, inhibition of AMPK with Compound C abolishes the negative effect of NaHS on global matrix protein synthesis induced by high glucose [157], implying the necessary role of AMPK in H_2_S-mediated effects on diabetic renal disease. Intriguingly, induction of inducible nitric oxide synthase (iNOS), not endothelial nitric oxide synthase (eNOS), is required for H_2_S to block high glucose-induced oxidative stress and matrix protein overproduction in renal proximal tubular epithelial cells, suggesting a role of NO in H_2_S-mediated beneficial effects against diabetic kidney disease [49]. These two gasotransmitters H_2_S and NO may be considered as therapeutic targets in diabetic nephropathy (Figure 6).

## 7. Glomerular Expansion and H_2_S in Diabetic Kidney Disease

Glomerular mesangial cells are vital components that maintain the normal morphology and functions of glomeruli [158]. Under diabetic conditions, both cell proliferation and ECM production of glomerular mesangial cells are increased, and those aberrant changes will ultimately lead to the thickening of glomerular basal membrane, and glomerular expansion [5,159,160]. Diabetic nephropathy is reflected by the abnormal ECM production and the phenotypic change of glomerular mesangial cells [161]. Data suggest that numerous glomerular diseases are associated with glomerular expansion, which may eventually produce glomerular scarring, especially in diabetes [162].

**Figure 7:**
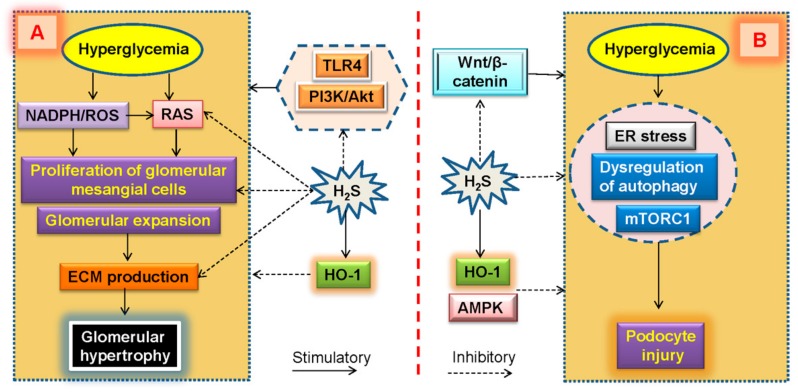
Effect of H_2_S on glomerular hypertrophy and podocyte injury in diabetic kidneys. (**A**) Activation of intrarenal renin-angiotensin system and NADPH-derived ROS contribute to the proliferation and ECM secretion in high glucose-incubated renal mesangial cells, this phenomenon is attenuated by H_2_S, dependent on HO-1 induction and inhibition of TLR4 and PI3K/Akt pathway. (**B**) Further studies reveal that endoplasmic reticulum stress, dysregulation of autophagy and mTORC1 activation in podocyte promote the development of diabetic nephropathy. H_2_S may induce AMPK phosphorylation and HO-1, and suppress the Wnt/β-catenin pathway to mitigate podocyte injury induced by hyperglycemia. NADPH, reduced nicotinamide adenine dinucleotide phosphate; ROS, reactive oxygen species; ECM, extracellular matrix; HO-1, heme oxygenase-1; TLR4, toll-like receptor 4; mTORC1, mammalian target of rapamycin (mTOR) complex 1; AMPK, adenosine 5’-monophosphate (AMP)-activated protein kinase.

Over-activation of intrarenal RAS is known as a crucial step in the pathogenesis of diabetic nephropathy [163]. Within the RAS, Ang II is one of the biologically active peptides of RAAS, and it is also regarded as an essential mediator for diabetic nephropathy [164,165]. Activation of intrarenal RAS has been implicated in glomerular enlargement and secondary glomerulosclerosis, interstitial fibroblast proliferation and ECM deposition [163,166]. In glomerulus, activated RAS may result in the proliferation of glomerular mesangial cells [167]. It has been revealed that high glucose stimulates ROS production and promotes fibronectin and collagen IV synthesis in cultured renal mesangial cells [168]. Exposure to high glucose raises the proliferation of rat renal glomerular mesangial cells, along with decreased the CSE expression and H_2_S level, whereas supplement of H_2_S prevents high glucose-triggered proliferation and ECM secretion in rat mesangial cells [45]. Mechanistically, inactivation of intrarenal RAS by H_2_S is requisite for inhibition of cell proliferation rate and TGF-β1 and of collagen IV productions in renal mesangial cells [86]. This effect may be dependent on attenuation of ROS generation because administration of NADPH oxidase inhibitor is capable of reversing the hyperglycemia-induced RAS activation and the following cell proliferation as well as collagen synthesis of renal mesangial cells [86]. After treatment with the H_2_S donors in human mesangial cells, the induction of antioxidant enzyme HO-1 may be a potential mechanism whereby H_2_S exerts its protective effects in the context of high glucose [169]. Additionally, stimulation of high glucose facilitates the proliferation of mesangial cells, which is coupled with reduced endogenous H_2_S synthesis. Exogenous H_2_S markedly mitigates high glucose-induced over-proliferation of mouse mesangial cells via inhibition of toll-like receptor 4 (TLR4) and PI3K/Akt pathway [170]. Collectively, the decreased H_2_S level responses to high glucose results in mesangial cell proliferation, which, in turn, contributes to the pathogenesis of diabetic glomerular hypertrophy and renal dysfunction (Figure 7).

## 8. Podocyte Injury and H_2_S in Diabetic Kidney Disease

Over the past decade, overwhelming evidence has pointed to the podocyte as a key target of renal injury during diabetic kidney disease progression [171,172,173,174]. Once the RAS is activated in diabetic kidneys, it may induce the generation of growth factors or cytokines such as TGF-β, vascular endothelial growth factor (VEGF), and monocyte chemotactic protein 1 (MCP-1), which may directly or indirectly lead to renal fibrosis, oxidative stress and podocyte apoptosis [175]. Studies from diabetic patients and animal models have revealed that the onset of albuminuria is closely related with podocyte injury, such as podocyte hypertrophy, detachment, apoptosis and foot process effacement [173]. Experimental data support that podocytes become nephrin-absent, effaced, and apoptotic, these events are correlated with the emergence of albuminuria [176]. Generally, the effacement and loss of podocytes are key events in the early progression of diabetic kidney disease [177]. AGEs dose- and time-dependently induce the apoptosis in murine podocytes via endoplasmic reticulum stress-mediated apoptotic pathway [178]. Further studies establish that dysregulation of autophagy or mTORC1 activation in podocytes participates in the development of diabetic nephropathy [179,180,181]. According to previous studies, the contribution of podocyte injury to diabetic nephropathy is a subject of great interest.

High glucose significantly reduced CSE expression in cultured mouse podocytes, and this podocyte injury responses to high glucose is alleviated by exogenous H_2_S possibly through Zona occludens-2 (ZO-2) upregulation and the subsequent suppression of Wnt/β-catenin pathway [182]. Moreover, it is demonstrated a significant increase in HO-1 expression after incubation with the H_2_S donors in both mesangial and podocyte cells [169], hinting that the ability to promote antioxidant enzyme HO-1 expression might be a potential mechanism by which H_2_S exerts its protective effects (Figure 7). Additional studies are needed to better recognize the molecular mechanisms of H_2_S in diabetic podocyte injury, and these studies could lead to novel therapeutic strategies for diabetic nephropathy.

## 9. Phytopharmaceuticals/Agents-Mediated H_2_S Induction in Diabetic Kidney Disease

As discussed above, H_2_S donors such as NaHS or GYY4137 might be a good therapeutic strategy for the treatment of diabetic kidney disease (Table 1 and Table 2). However, recent findings have disclosed that some phytopharmaceuticals or agents that participate with H_2_S may exhibit renal protective effects in diabetes (Table 3). A novel H_2_S-releasing compound, S-propargyl-cysteine, attenuates inflammation in diabetic kidneys via suppressing the phosphorylation of extracellular regulated protein kinases (ERK), p38 protein [183]. Tadalafil abrogates the global protein synthesis and matrix protein laminin γ1 in kidney podocytes induced by high glucose, this may be relied on induction of H_2_S [184]. Induction of CSE-derived H_2_S by tadalafil accelerates AMPK phosphorylation by stimulating calcium-calmodulin kinase kinase β, followed by inhibition of mTORC1 activity and mRNA translation in high glucose-treated kidney podocytes [184]. Metformin, an anti-diabetic agent, is widely prescribed to treat diabetic kidney disorder through saving the podocytes [185], this effect may be related with progressively increased H_2_S concentration in the kidney [186]. Together with these results, it is reasonable to recommend that the induction of H_2_S by natural plant-derived compounds might be potential therapeutic candidates to ameliorate diabetic nephropathy. However, more investigation on this subject is necessary.

## 10. Current Molecular Mechanisms of H_2_S in Diabetic Kidney Disease

H_2_S has recently gained enormous attention in diabetic nephropathy since its critical role in the progression of diabetic nephropathy [7,188]. Circulating H_2_S levels appear to be reduced in type 2 diabetic subjects [46], and are inversely correlated with adiposity parameters [47]. Animal studies have highlighted the crucial role of H_2_S dysregulation in diabetic kidney disease. Sen and colleagues have revealed that induction of matrix metalloproteinase-9 (MMP-9) elicits the decreased production of H_2_S-synthesizing enzymes CBS and CSE in the diabetic kidney [130]. The same group further demonstrates that H_2_S donor GYY4137 ameliorates diabetic oxidative injury and renal fibrosis through miR-194-dependent pathway [187]. The downregulated miR-194 is restored by H_2_S, followed by diminished ROS production, and normalized MMP-9, MMP-13 and MMP-14 in glomerular endothelial cells under high glucose condition [187]. TGF-β is critically involved in the synthesis of matrix proteins and diabetic renal fibrosis [189,190]. The increased expressions of TGF-β and matrix proteins as well as urinary albumin loss in diabetic mice are reversed by H_2_S administration [45,50]. Additionally, the enhanced matrix protein synthesis is a contributor for renal hypertrophy [191]. It is well documented that PI3K-Akt-mammalian target of rapamycin (mTOR) signaling pathway plays a key role in matrix protein synthesis during diabetic kidney disease [7], since blockade of this pathway ameliorates renal injury in diabetic rodent models [192]. An additional signaling change in diabetic kidney is reduced activity of 5’-monophosphate (AMP)-activated protein kinase (AMPK), which is indicated to abrogate diabetic kidney injury [193,194]. In high glucose-treated renal epithelial cells, H_2_S donor NaHS stimulates AMPK activity, reduces mTOR complex-1 (mTORC1) activity and prevents matrix protein deposition [157]. NaHS also retards high glucose-induced mesangial proliferation through inhibition of mitogen-activated protein kinases (MAPK) activity [50]. The existing data demonstrate that the important signaling pathways recruited by H_2_S are sufficient to ameliorate diabetic nephropathy. However, more research is required to elucidate additional mechanisms by which H_2_S exerts a protective effect in diabetic kidney injury. Next, in detail, we will outline the current studies about the interactions between dysregulated H_2_S and other renal lesions in diabetic kidney disease.

## 11. Conclusions and Perspectives

The novel and constructive insights into progressive diabetic nephropathy become urgent due to the continuously increasing incidence of diabetes and obesity. Continuous experimental studies have been performed to identify the possible molecular mediators in diabetic kidney disease. As the third gaseous mediator after NO and CO, the H_2_S signaling pathway may provide potential therapeutic target for the treatment of diabetic kidney disease. Targeting the H_2_S signaling pathway may be postulated to be a reprogramming strategy against diabetic kidney disease. However, many questions remain regarding which renal cells are most affected by decreased H_2_S level and why the renal H_2_S level is downregulated in response to hyperglycemia? As of yet, few studies evaluate the temporal patterns of H_2_S activation and it is probable that timing will have a profound effect on outcomes. Focused studies investigating the roles of H_2_S in humans or rodent models will broaden our understanding of the H_2_S signaling pathway in diabetic kidney disease.

Based on the underlying mechanisms whereby H_2_S exerts protective effects against diabetic kidney disease, the precursors for H_2_S synthesis, H_2_S donors, and natural plant-derived compounds for H_2_S generation are indicated to ameliorate tubular lesions, and reverse renal injury in diabetes. However, the challenge still exists, the regression of renal lesions and recovery of renal structure should be the focus in future novel therapeutic strategies. Similarly, it should be mentioned that the possible molecular mechanisms of H_2_S in diabetic nephropathy are not fully understood. Thereafter, in-depth understanding of interaction between H_2_S and its downstream targeted genes will undoubtedly help to propel the development of H_2_S-medaited novel therapies that can halt or reverse diabetic renal disease. Moreover, more research is required to understand how H_2_S dysfunction interacted with other pathogenic factors in diabetic kidney disease.

A wide range of impressive studies focusing the role of H_2_S in diabetic renal damage are growing rapidly in recent years. Accordingly, the expressions of H_2_S-producing enzymes and levels of its precursors and metabolites are abnormal in diabetic renal pathology. With this in mind, it is interesting to know whether H_2_S or its metabolites are thought to be biomarkers for renal disease severity in diabetes. Nevertheless, only a few studies have addressed this uncertainty. To answer this possibility, large population studies investigating the clinical value of H_2_S metabolites as predictors of diabetic kidney disease are underway.

The emerging approach that is highly relevant to the H_2_S signaling pathway is a flourishing field because of the increasing epidemic of diabetic kidney disease. However, these results await further clinical translation. N-acetylcysteine (NAC) is a derivate of cysteine and it is the main substrate for H_2_S production [195]. Therefore, NAC may be able to promote H_2_S production in humans. For this reason, a clinical trial had investigated the effect of NAC on the production of H_2_S in patients with CKD several years ago (ClinicalTrials.gov Identifier: NCT01232257). However, to date, it is still unknown whether NAC could increase H_2_S levels in CKD patients or dialysis patients. As such, more large-scale clinical studies are needed to further determine the protective role of H_2_S in kidney diseases including diabetic nephropathy.

Taken together, since the impaired H_2_S signaling pathway appears to be one of the denominators for diabetic renal injury, it may be a suitable target to ameliorate renovascular complications of diabetes. With more and more excited discoveries regarding H_2_S functions in renal physiology and disease, we expect multiple innovative H_2_S applications to evolve in the near future.

## Figures and Tables

**Table 1:** Beneficial effect of H_2_S on experimental diabetic nephropathy in vitro.

H_2_S Donors	Cell Type	Main Findings	Ref.
NaHS	Renal fibroblasts	H_2_S inhibits the proliferation of renal fibroblasts. Furthermore, the differentiation of quiescent renal fibroblasts to myofibroblasts is prevented by H_2_S, which involves the inhibition of TGF-β1-Smad and MAPK signaling pathways.	[38]
NaHS	Glomerular mesangial cells	NaHS inhibits the ROS generation and cell proliferation, and downregulates the expressions of TGF-β1 and collagen IV in high glucose-incubated cells.	[45]
NaHS	Renal tubular epithelial cells.	The activation of AMPK by H_2_S prevents high glucose-induced NOX4 expression in epithelial cells. NaHS augments the expression of iNOS, this effect is involved in the protective effect of H_2_S against high glucose-induced NOX4 expression, ROS generation, and matrix laminin expression.	[49]
NaHS	Glomerular mesangial cells	H_2_S activates the Nrf2 signaling pathway to restrain high glucose-induced oxidative stress. H_2_S exerts anti-inflammatory effects by blocking NF-κB signaling. Additionally, the cell proliferation induced by high glucose is mediated by MAPK signaling pathways, which is impeded by H_2_S.	[50]
NaHS	Glomerular mesangial cells	Supplementation of H_2_S represses the cell proliferation, inhibits TGF-β1 and collagen IV expressions, and attenuates the elevation of ROS in high glucose-treated cells. Meanwhile, AGT, ACE and AT1 receptor mRNA levels and Ang II concentration are upregulated in high glucose-challenged cells, which are diminished by H_2_S.	[86]
NaHS	Renal tubular epithelial cells	Blockade of ERK- and β-catenin-dependent pathways may be involved in the suppressive effect of H_2_S on TGF-β1-induced renal EMT in renal tubular epithelial cells, as evidenced by upregulated levels of E-cadherin, along with downregulated expressions of α-SMA and fibronectin.	[156]
NaHS	Renal tubular epithelial cells	The activation of mTORC1 and inactivation of AMPK are involved in global matrix protein synthesis, and these events are all reversed by NaHS. Importantly, NaHS stimulates AMPK phosphorylation and restores AMPK phosphorylation induced by high glucose.	[157]
AP39, AP106, AP72, AP67, GYY4134	Glomerular mesangial cells, podocytes	H_2_S upregulates the expression of HO-1 in both mesangial and podocyte cells. H_2_S might have the ability to upregulate this antioxidant enzyme, which may be a potential mechanism by which H_2_S exerts its protective effects.	[169]
NaHS	Glomerular mesangial cells	Exogenous H_2_S treatment mitigates the proliferation of mesangial cells. Furthermore, H_2_S supplementation remarkably inhibits TLR4 expression and curbs the mesangial cell proliferation.	[170]
NaHS	Mouse podocytes	High glucose stimulation significantly reduces nephrin, ZO-2, and CSE expression levels, and elevates β-catenin production in mouse podocytes. Supplementation of NaHS rectifies these changes. Exogenous H_2_S may alleviate high glucose-induced podocyte injury possibly through ZO-2 upregulation and the subsequent suppression of Wnt/β-catenin pathway.	[182]
GYY4137	Glomerular endothelial cells	GYY4137 upregulates miR-194 level to mitigate ROS production under high glucose condition.	[187]

**Table 2:** Beneficial effect of H_2_S on experimental diabetic nephropathy in vivo.

H_2_S Donors	Animal Models	Main Findings	Ref.
NaHS	STZ-induced diabetic rats	Administration of NaHS reverses the increases in TGF-β1 and collagen IV in diabetic rats.	[45]
NaHS	STZ-induced diabetic rats	H_2_S attenuates glomerular basement membrane thickening, mesangial matrix deposition, and renal interstitial fibrosis, thereby improving improve renal function in diabetic rats. The protein expressions of ACE and AT1 receptors as well as Ang II are significantly up-regulated in diabetic kidneys and down-regulated after treatment with H_2_S.	[50]
NaHS	STZ-induced diabetic rats	In STZ-induced diabetic rats, the changes in RAS are reversed by H_2_S supplementation without affecting blood glucose concentration.	[86]
NaHS	STZ-induced diabetic rats	The increased 24 h urinary protein, fasting blood glucose (FBG), blood urea nitrogen (BUN), serum creatinine (Scr) and renal index, as well as the elevated amount of glomerular mesangial matrix in diabetic rats are all ameliorated by H_2_S treatment. In addition, the diabetic kidney shows the increased MDA content, caspase-3 activity and Bax expression, but decreased SOD activity and BCl^−^2 expression, which are normalized by administration of H_2_S.	[95]
NaHS	Mice with type 1 diabetes or type 2 diabetes	Renal cortical contents of CBS and CSE are significantly reduced, alongside with renal hypertrophy and matrix accumulation in mice with type 1 diabetes or type 2 diabetes.	[157]
GYY4137	Diabetic Akita mice	GYY4137 prevents collagen deposition and realignment and renal fibrosis in mice. The increased expressions of MMP-9, MMP-13 and MMP-14, and reduced vascular density in diabetic kidney are reversed by GYY4137.	[187]

**Table 3:** Beneficial effect of H_2_S-releasing compounds on experimental diabetic nephropathy.

H_2_S-Releasing Compounds	Animal Models/Cell Type	Main Finding	Ref.
S-propargyl-cysteine	STZ-induced diabetic rat/mesangial cells	S-propargyl-cysteine, a H_2_S-releasing compound, reduces the level of creatinine, kidney to body weight ratio and 24-h urine microalbuminuria excretion in STZ-induced diabetic kidney injury. The renal fibrosis, inflammation, and hypertrophy are suppressed by this compound. The renal protective effects of this compound may be mediated by inhibition of TGF-β1/Smad3 pathway and blockade of MAPK signaling pathway.	[183]
Tadalafil	Podocytes	Tadalafil, increases the expression and activity of the H_2_S-generating enzyme CSE by accelerating its translation. It can effectively abrogate high glucose-induced global protein synthesis in podocytes. Tadalafil activates AMPK by stimulating calcium-calmodulin kinase β, thus attenuating the activation of mTOR induced by high glucose. Furthermore, in tadalafil-treated podocytes, the iNOS expression is rapidly upregulated. Knockdown or inhibition of iNOS abolished the effect of tadalafil on CSE expression and AMPK phosphorylation in podocytes.	[184]

## References

[1] Duran-Salgado M.B., Rubio-Guerra A.F. (2014). Diabetic nephropathy and inflammation. World J. Diabetes.

[2] Choi B.H., Kang K.S., Kwak M.K. (2014). Effect of redox modulating NRF2 activators on chronic kidney disease. Molecules.

[3] Feliers D., Lee H.J., Kasinath B.S. (2016). Hydrogen sulfide in renal physiology and disease. Antioxid. Redox Signal..

[4] Afkarian M., Sachs M.C., Kestenbaum B., Hirsch I.B., Tuttle K.R., Himmelfarb J., de Boer I.H. (2013). Kidney disease and increased mortality risk in type 2 diabetes. J. Am. Soc. Nephrol. JASN.

[5] Gruden G., Perin P.C., Camussi G. (2005). Insight on the pathogenesis of diabetic nephropathy from the study of podocyte and mesangial cell biology. Curr. Diabetes Rev..

[6] Dugbartey G.J. (2017). Diabetic nephropathy: A potential savior with ‘rotten-egg’ smell. Pharmacol. Rep. PR.

[7] Kasinath B.S., Feliers D., Lee H.J. (2018). Hydrogen sulfide as a regulatory factor in kidney health and disease. Biochem. Pharmacol..

[8] Cao X., Bian J.S. (2016). The role of hydrogen sulfide in renal system. Front. Pharmacol..

[9] Kashfi K. (2018). The role of hydrogen sulfide in health and disease. Biochem. Pharmacol..

[10] Cao X., Zhang W., Moore P.K., Bian J. (2019). Protective smell of hydrogen sulfide and polysulfide in cisplatin-induced nephrotoxicity. Int. J. Mol. Sci..

[11] Koning A.M., Frenay A.R., Leuvenink H.G., van Goor H. (2015). Hydrogen sulfide in renal physiology, disease and transplantation—The smell of renal protection. Nitric Oxide Biol. Chem..

[12] Lobb I., Sonke E., Aboalsamh G., Sener A. (2015). Hydrogen sulphide and the kidney: Important roles in renal physiology and pathogenesis and treatment of kidney injury and disease. Nitric Oxide Biol. Chem..

[13] Alicic R.Z., Rooney M.T., Tuttle K.R. (2017). Diabetic kidney disease: Challenges, progress, and possibilities. Clin. J. Am. Soc. Nephrol. CJASN.

[14] Lin Y.C., Chang Y.H., Yang S.Y., Wu K.D., Chu T.S. (2018). Update of pathophysiology and management of diabetic kidney disease. J. Formos. Med Assoc. Taiwan Yi Zhi.

[15] Tuttle K.R. (2017). Back to the future: Glomerular hyperfiltration and the diabetic kidney. Diabetes.

[16] Potenza M.A., Gagliardi S., Nacci C., Carratu M.R., Montagnani M. (2009). Endothelial dysfunction in diabetes: From mechanisms to therapeutic targets. Curr. Med. Chem..

[17] De Zeeuw D., Coll B., Andress D., Brennan J.J., Tang H., Houser M., Correa-Rotter R., Kohan D., Lambers Heerspink H.J., Makino H. (2014). The endothelin antagonist atrasentan lowers residual albuminuria in patients with type 2 diabetic nephropathy. J. Am. Soc. Nephrol. JASN.

[18] Prabhakar S.S. (2004). Role of nitric oxide in diabetic nephropathy. Semin. Nephrol..

[19] Bernhardt W.M., Schmitt R., Rosenberger C., Munchenhagen P.M., Grone H.J., Frei U., Warnecke C., Bachmann S., Wiesener M.S., Willam C. (2006). Expression of hypoxia-inducible transcription factors in developing human and rat kidneys. Kidney Int..

[20] Giacchetti G., Sechi L.A., Rilli S., Carey R.M. (2005). The renin-angiotensin-aldosterone system, glucose metabolism and diabetes. Trends Endocrinol. Metab. TEM.

[21] Toma I., Kang J.J., Sipos A., Vargas S., Bansal E., Hanner F., Meer E., Peti-Peterdi J. (2008). Succinate receptor GPR91 provides a direct link between high glucose levels and renin release in murine and rabbit kidney. J. Clin. Investig..

[22] Gurley S.B., Coffman T.M. (2007). The renin-angiotensin system and diabetic nephropathy. Semin. Nephrol..

[23] He J., Xu Y., Koya D., Kanasaki K. (2013). Role of the endothelial-to-mesenchymal transition in renal fibrosis of chronic kidney disease. Clin. Exp. Nephrol..

[24] Ritz E., Tomaschitz A. (2009). Aldosterone, a vasculotoxic agent—Novel functions for an old hormone. Nephrol. Dial. Transplant. Off. Publ. Eur. Dial. Transplant. Assoc. Eur. Ren. Assoc..

[25] Ziyadeh F.N., Wolf G. (2008). Pathogenesis of the podocytopathy and proteinuria in diabetic glomerulopathy. Curr. Diabetes Rev..

[26] Brosius F.C., Tuttle K.R., Kretzler M. (2016). JAK inhibition in the treatment of diabetic kidney disease. Diabetologia.

[27] Wang R. (2002). Two’s company, three’s a crowd: Can H_2_S be the third endogenous gaseous transmitter?. FASEB J. Off. Publ. Fed. Am. Soc. Exp. Biol..

[28] Li L., Hsu A., Moore P.K. (2009). Actions and interactions of nitric oxide, carbon monoxide and hydrogen sulphide in the cardiovascular system and in inflammation–A tale of three gases!. Pharmacol. Ther..

[29] Mathai J.C., Missner A., Kugler P., Saparov S.M., Zeidel M.L., Lee J.K., Pohl P. (2009). No facilitator required for membrane transport of hydrogen sulfide. Proc. Natl. Acad. Sci. USA.

[30] Paul B.D., Snyder S.H. (2012). H(2)S signalling through protein sulfhydration and beyond. Nat. Rev. Mol. Cell Biol..

[31] Singh S., Padovani D., Leslie R.A., Chiku T., Banerjee R. (2009). Relative contributions of cystathionine beta-synthase and gamma-cystathionase to H_2_S biogenesis via alternative trans-sulfuration reactions. J. Biol. Chem..

[32] Kimura H. (2014). Hydrogen sulfide and polysulfides as biological mediators. Molecules.

[33] Modis K., Coletta C., Erdelyi K., Papapetropoulos A., Szabo C. (2013). Intramitochondrial hydrogen sulfide production by 3-mercaptopyruvate sulfurtransferase maintains mitochondrial electron flow and supports cellular bioenergetics. FASEB J. Off. Publ. Fed. Am. Soc. Exp. Biol..

[34] Tanizawa K. (2011). Production of H_2_S by 3-mercaptopyruvate sulphurtransferase. J. Biochem..

[35] Liu Y.H., Lu M., Hu L.F., Wong P.T., Webb G.D., Bian J.S. (2012). Hydrogen sulfide in the mammalian cardiovascular system. Antioxid. Redox Signal..

[36] Shibuya N., Koike S., Tanaka M., Ishigami-Yuasa M., Kimura Y., Ogasawara Y., Fukui K., Nagahara N., Kimura H. (2013). A novel pathway for the production of hydrogen sulfide from D-cysteine in mammalian cells. Nat. Commun..

[37] Kamoun P. (2004). Endogenous production of hydrogen sulfide in mammals. Amino Acids.

[38] Song K., Wang F., Li Q., Shi Y.B., Zheng H.F., Peng H., Shen H.Y., Liu C.F., Hu L.F. (2014). Hydrogen sulfide inhibits the renal fibrosis of obstructive nephropathy. Kidney Int..

[39] Zhang S., Pan C., Zhou F., Yuan Z., Wang H., Cui W., Zhang G. (2015). Hydrogen sulfide as a potential therapeutic target in fibrosis. Oxid. Med. Cell. Longev..

[40] Bos E.M., Wang R., Snijder P.M., Boersema M., Damman J., Fu M., Moser J., Hillebrands J.L., Ploeg R.J., Yang G. (2013). Cystathionine gamma-lyase protects against renal ischemia/reperfusion by modulating oxidative stress. J. Am. Soc. Nephrol. JASN.

[41] Xia M., Chen L., Muh R.W., Li P.L., Li N. (2009). Production and actions of hydrogen sulfide, a novel gaseous bioactive substance, in the kidneys. J. Pharmacol. Exp. Ther..

[42] Wang R. (2012). Physiological implications of hydrogen sulfide: A whiff exploration that blossomed. Physiol. Rev..

[43] Kabil O., Vitvitsky V., Xie P., Banerjee R. (2011). The quantitative significance of the transsulfuration enzymes for H_2_S production in murine tissues. Antioxid. Redox Signal..

[44] Xue R., Hao D.D., Sun J.P., Li W.W., Zhao M.M., Li X.H., Chen Y., Zhu J.H., Ding Y.J., Liu J. (2013). Hydrogen sulfide treatment promotes glucose uptake by increasing insulin receptor sensitivity and ameliorates kidney lesions in type 2 diabetes. Antioxid. Redox Signal..

[45] Yuan P., Xue H., Zhou L., Qu L., Li C., Wang Z., Ni J., Yu C., Yao T., Huang Y. (2011). Rescue of mesangial cells from high glucose-induced over-proliferation and extracellular matrix secretion by hydrogen sulfide. Nephrol. Dial. Transplant. Off. Publ. Eur. Dial. Transplant. Assoc. Eur. Ren. Assoc..

[46] Jain S.K., Bull R., Rains J.L., Bass P.F., Levine S.N., Reddy S., McVie R., Bocchini J.A. (2010). Low levels of hydrogen sulfide in the blood of diabetes patients and streptozotocin-treated rats causes vascular inflammation?. Antioxid. Redox Signal..

[47] Whiteman M., Gooding K.M., Whatmore J.L., Ball C.I., Mawson D., Skinner K., Tooke J.E., Shore A.C. (2010). Adiposity is a major determinant of plasma levels of the novel vasodilator hydrogen sulphide. Diabetologia.

[48] Li H., Feng S.J., Zhang G.Z., Wang S.X. (2014). Correlation of lower concentrations of hydrogen sulfide with atherosclerosis in chronic hemodialysis patients with diabetic nephropathy. Blood Purif..

[49] Lee H.J., Lee D.Y., Mariappan M.M., Feliers D., Ghosh-Choudhury G., Abboud H.E., Gorin Y., Kasinath B.S. (2017). Hydrogen sulfide inhibits high glucose-induced NADPH oxidase 4 expression and matrix increase by recruiting inducible nitric oxide synthase in kidney proximal tubular epithelial cells. J. Biol. Chem..

[50] Zhou X., Feng Y., Zhan Z., Chen J. (2014). Hydrogen sulfide alleviates diabetic nephropathy in a streptozotocin-induced diabetic rat model. J. Biol. Chem..

[51] Ge S.N., Zhao M.M., Wu D.D., Chen Y., Wang Y., Zhu J.H., Cai W.J., Zhu Y.Z., Zhu Y.C. (2014). Hydrogen sulfide targets EGFR Cys797/Cys798 residues to induce Na (+)/K (+)-ATPase endocytosis and inhibition in renal tubular epithelial cells and increase sodium excretion in chronic salt-loaded rats. Antioxid. Redox Signal..

[52] Zhang J., Chen S., Liu H., Zhang B., Zhao Y., Ma K., Zhao D., Wang Q., Ma H., Zhang Z. (2013). Hydrogen sulfide prevents hydrogen peroxide-induced activation of epithelial sodium channel through a PTEN/PI (3,4,5)P3 dependent pathway. PLoS ONE.

[53] Agne A.M., Baldin J.P., Benjamin A.R., Orogo-Wenn M.C., Wichmann L., Olson K.R., Walters D.V., Althaus M. (2015). Hydrogen sulfide decreases beta-adrenergic agonist-stimulated lung liquid clearance by inhibiting ENaC-mediated transepithelial sodium absorption. Am. J. Physiol. Regul. Integr. Comp. Physiol..

[54] Liu Y.H., Bian J.S. (2010). Bicarbonate-dependent effect of hydrogen sulfide on vascular contractility in rat aortic rings. Am. J. Physiol. Cell Physiol..

[55] Lee E.J., Hyun S.H., Chun J., Kang S.S. (2007). Human NIMA-related kinase 6 is one of the Fe65 WW domain binding proteins. Biochem. Biophys. Res. Commun..

[56] Olson K.R. (2015). Hydrogen sulfide as an oxygen sensor. Antioxid. Redox Signal..

[57] Kumar P., Prabhakar N.R. (2012). Peripheral chemoreceptors: Function and plasticity of the carotid body. Compr. Physiol..

[58] Olson K.R., Dombkowski R.A., Russell M.J., Doellman M.M., Head S.K., Whitfield N.L., Madden J.A. (2006). Hydrogen sulfide as an oxygen sensor/transducer in vertebrate hypoxic vasoconstriction and hypoxic vasodilation. J. Exp. Biol..

[59] Olson K.R., Whitfield N.L. (2010). Hydrogen sulfide and oxygen sensing in the cardiovascular system. Antioxid. Redox Signal..

[60] Hu H., Shi Y., Chen Q., Yang W., Zhou H., Chen L., Tang Y., Zheng Y. (2008). Endogenous hydrogen sulfide is involved in regulation of respiration in medullary slice of neonatal rats. Neuroscience.

[61] Dombkowski R.A., Naylor M.G., Shoemaker E., Smith M., DeLeon E.R., Stoy G.F., Gao Y., Olson K.R. (2011). Hydrogen sulfide (H_2_S) and hypoxia inhibit salmonid gastrointestinal motility: Evidence for H_2_S as an oxygen sensor. J. Exp. Biol..

[62] Hirakawa Y., Tanaka T., Nangaku M. (2017). Renal hypoxia in CKD; Pathophysiology and detecting methods. Front. Physiol..

[63] Teng H., Wu B., Zhao K., Yang G., Wu L., Wang R. (2013). Oxygen-sensitive mitochondrial accumulation of cystathionine beta-synthase mediated by Lon protease. Proc. Natl. Acad. Sci. USA.

[64] Fu M., Zhang W., Wu L., Yang G., Li H., Wang R. (2012). Hydrogen sulfide (H_2_S) metabolism in mitochondria and its regulatory role in energy production. Proc. Natl. Acad. Sci. USA.

[65] Beltowski J. (2010). Hypoxia in the renal medulla: Implications for hydrogen sulfide signaling. J. Pharmacol. Exp. Ther..

[66] Kurtz A. (2017). Endocrine functions of the renal interstitium. Pflug. Arch. Eur. J. Physiol..

[67] Zeisberg M., Kalluri R. (2015). Physiology of the renal interstitium. Clin. J. Am. Soc. Nephrol. CJASN.

[68] Tong L., Adler S.G. (2018). Diabetic kidney disease. Clin. J. Am. Soc. Nephrol. CJASN.

[69] Hostetter T.H., Troy J.L., Brenner B.M. (1981). Glomerular hemodynamics in experimental diabetes mellitus. Kidney Int..

[70] Anderson S., Meyer T.W., Rennke H.G., Brenner B.M. (1985). Control of glomerular hypertension limits glomerular injury in rats with reduced renal mass. J. Clin. Investig..

[71] Zatz R., Dunn B.R., Meyer T.W., Anderson S., Rennke H.G., Brenner B.M. (1986). Prevention of diabetic glomerulopathy by pharmacological amelioration of glomerular capillary hypertension. J. Clin. Investig..

[72] Bermejo S., Garcia C.O., Rodriguez E., Barrios C., Otero S., Mojal S., Pascual J., Soler M.J. (2018). The renin-angiotensin-aldosterone system blockade in patients with advanced diabetic kidney disease. Nefrologia.

[73] Hsueh W.A. (2002). Treatment of type 2 diabetic nephropathy by blockade of the renin-angiotensin system: A comparison of angiotensin-converting-enzyme inhibitors and angiotensin receptor antagonists. Curr. Opin. Pharmacol..

[74] Jacobsen P.K. (2005). Preventing end-stage renal disease in diabetic patients—Dual blockade of the renin-angiotensin system (Part II). J. Renin Angiotensin Aldosterone Syst. JRAAS.

[75] Sarafidis P.A., Stafylas P.C., Kanaki A.I., Lasaridis A.N. (2008). Effects of renin-angiotensin system blockers on renal outcomes and all-cause mortality in patients with diabetic nephropathy: An updated meta-analysis. Am. J. Hypertens..

[76] Schweda F., Friis U., Wagner C., Skott O., Kurtz A. (2007). Renin release. Physiology.

[77] Lim J.J., Liu Y.H., Khin E.S., Bian J.S. (2008). Vasoconstrictive effect of hydrogen sulfide involves downregulation of cAMP in vascular smooth muscle cells. Am. J. Physiol. Cell Physiol..

[78] Yong Q.C., Pan T.T., Hu L.F., Bian J.S. (2008). Negative regulation of beta-adrenergic function by hydrogen sulphide in the rat hearts. J. Mol. Cell. Cardiol..

[79] Lu M., Liu Y.H., Goh H.S., Wang J.J., Yong Q.C., Wang R., Bian J.S. (2010). Hydrogen sulfide inhibits plasma renin activity. J. Am. Soc. Nephrol. JASN.

[80] Liu Y.H., Lu M., Xie Z.Z., Hua F., Xie L., Gao J.H., Koh Y.H., Bian J.S. (2014). Hydrogen sulfide prevents heart failure development via inhibition of renin release from mast cells in isoproterenol-treated rats. Antioxid. Redox Signal..

[81] Li Z., Organ C.L., Kang J., Polhemus D.J., Trivedi R.K., Sharp T.E., Jenkins J.S., Tao Y.X., Xian M., Lefer D.J. (2018). Hydrogen sulfide attenuates renin angiotensin and aldosterone pathological signaling to preserve kidney function and improve exercise tolerance in heart failure. JACC Basic Transl. Sci..

[82] Kennefick T.M., Anderson S. (1997). Role of angiotensin II in diabetic nephropathy. Semin. Nephrol..

[83] Wolf G., Butzmann U., Wenzel U.O. (2003). The renin-angiotensin system and progression of renal disease: From hemodynamics to cell biology. Nephron. Physiol..

[84] Zhuo J.L., Li X.C. (2011). New insights and perspectives on intrarenal renin-angiotensin system: Focus on intracrine/intracellular angiotensin II. Peptides.

[85] Sonkodi S., Mogyorosi A. (2003). Treatment of diabetic nephropathy with angiotensin II blockers. Nephrol. Dial. Transplant..

[86] Xue H., Yuan P., Ni J., Li C., Shao D., Liu J., Shen Y., Wang Z., Zhou L., Zhang W. (2013). H_2_S inhibits hyperglycemia-induced intrarenal renin-angiotensin system activation via attenuation of reactive oxygen species generation. PLoS ONE.

[87] Kashihara N., Haruna Y., Kondeti V.K., Kanwar Y.S. (2010). Oxidative stress in diabetic nephropathy. Curr. Med. Chem..

[88] LeBaron T.W., Kura B., Kalocayova B., Tribulova N., Slezak J. (2019). A new approach for the prevention and treatment of cardiovascular disorders. Molecular hydrogen significantly reduces the effects of oxidative stress. Molecules.

[89] Lee H.B., Yu M.R., Yang Y., Jiang Z., Ha H. (2003). Reactive oxygen species-regulated signaling pathways in diabetic nephropathy. J. Am. Soc. Nephrol. JASN.

[90] Ha H., Yu M.R., Choi Y.J., Kitamura M., Lee H.B. (2002). Role of high glucose-induced nuclear factor-kappaB activation in monocyte chemoattractant protein-1 expression by mesangial cells. J. Am. Soc. Nephrol. JASN.

[91] Ha H., Lee H.B. (2000). Reactive oxygen species as glucose signaling molecules in mesangial cells cultured under high glucose. Kidney Int. Suppl..

[92] Iglesias-De La Cruz M.C., Ruiz-Torres P., Alcami J., Diez-Marques L., Ortega-Velazquez R., Chen S., Rodriguez-Puyol M., Ziyadeh F.N., Rodriguez-Puyol D. (2001). Hydrogen peroxide increases extracellular matrix mRNA through TGF-beta in human mesangial cells. Kidney Int..

[93] Rhyu D.Y., Yang Y., Ha H., Lee G.T., Song J.S., Uh S.T., Lee H.B. (2005). Role of reactive oxygen species in TGF-beta1-induced mitogen-activated protein kinase activation and epithelial-mesenchymal transition in renal tubular epithelial cells. J. Am. Soc. Nephrol. JASN.

[94] Arora M.K., Singh U.K. (2014). Oxidative stress: Meeting multiple targets in pathogenesis of diabetic nephropathy. Curr. Drug Targets.

[95] Yang R., Liu X.F., Ma S.F., Gao Q., Li Z.H., Jia Q. (2016). Protective effect of hydrogen sulfide on kidneys of type 1 diabetic rats. Zhongguo Ying Yong Sheng Li Xue Za Zhi Zhongguo Yingyong Shenglixue Zazhi Chin. J. Appl. Physiol..

[96] Moi P., Chan K., Asunis I., Cao A., Kan Y.W. (1994). Isolation of NF-E2-related factor 2 (Nrf2), a NF-E2-like basic leucine zipper transcriptional activator that binds to the tandem NF-E2/AP1 repeat of the beta-globin locus control region. Proc. Natl. Acad. Sci. USA.

[97] Itoh K., Mimura J., Yamamoto M. (2010). Discovery of the negative regulator of Nrf2, Keap1: A historical overview. Antioxid. Redox Signal..

[98] Keum Y.S., Choi B.Y. (2014). Molecular and chemical regulation of the Keap1-Nrf2 signaling pathway. Molecules.

[99] Niture S.K., Khatri R., Jaiswal A.K. (2014). Regulation of Nrf2-an update. Free Radic. Biol. Med..

[100] Tong K.I., Katoh Y., Kusunoki H., Itoh K., Tanaka T., Yamamoto M. (2006). Keap1 recruits Neh2 through binding to ETGE and DLG motifs: Characterization of the two-site molecular recognition model. Mol. Cell. Biol..

[101] Itoh K., Wakabayashi N., Katoh Y., Ishii T., Igarashi K., Engel J.D., Yamamoto M. (1999). Keap1 represses nuclear activation of antioxidant responsive elements by Nrf2 through binding to the amino-terminal Neh2 domain. Genes Dev..

[102] Zhang D.D., Lo S.C., Cross J.V., Templeton D.J., Hannink M. (2004). Keap1 is a redox-regulated substrate adaptor protein for a Cul3-dependent ubiquitin ligase complex. Mol. Cell. Biol..

[103] Katoh Y., Itoh K., Yoshida E., Miyagishi M., Fukamizu A., Yamamoto M. (2001). Two domains of Nrf2 cooperatively bind CBP, a CREB binding protein, and synergistically activate transcription. Genes Cells Devoted Mol. Cell. Mech..

[104] McMahon M., Thomas N., Itoh K., Yamamoto M., Hayes J.D. (2004). Redox-regulated turnover of Nrf2 is determined by at least two separate protein domains, the redox-sensitive Neh2 degron and the redox-insensitive Neh6 degron. J. Biol. Chem..

[105] Wang H., Liu K., Geng M., Gao P., Wu X., Hai Y., Li Y., Li Y., Luo L., Hayes J.D. (2013). RXRalpha inhibits the NRF2-ARE signaling pathway through a direct interaction with the Neh7 domain of NRF2. Cancer Res..

[106] Hayes J.D., McMahon M. (2009). NRF2 and KEAP1 mutations: Permanent activation of an adaptive response in cancer. Trends Biochem. Sci..

[107] Katoh Y., Iida K., Kang M.I., Kobayashi A., Mizukami M., Tong K.I., McMahon M., Hayes J.D., Itoh K., Yamamoto M. (2005). Evolutionary conserved N-terminal domain of Nrf2 is essential for the Keap1-mediated degradation of the protein by proteasome. Arch. Biochem. Biophys..

[108] Saito R., Suzuki T., Hiramoto K., Asami S., Naganuma E., Suda H., Iso T., Yamamoto H., Morita M., Baird L. (2016). Characterizations of three major cysteine sensors of Keap1 in stress response. Mol. Cell. Biol..

[109] Wakabayashi N., Dinkova-Kostova A.T., Holtzclaw W.D., Kang M.I., Kobayashi A., Yamamoto M., Kensler T.W., Talalay P. (2004). Protection against electrophile and oxidant stress by induction of the phase 2 response: Fate of cysteines of the Keap1 sensor modified by inducers. Proc. Natl. Acad. Sci. USA.

[110] Ruiz S., Pergola P.E., Zager R.A., Vaziri N.D. (2013). Targeting the transcription factor Nrf2 to ameliorate oxidative stress and inflammation in chronic kidney disease. Kidney Int..

[111] Cui W., Bai Y., Luo P., Miao L., Cai L. (2013). Preventive and therapeutic effects of MG132 by activating Nrf2-ARE signaling pathway on oxidative stress-induced cardiovascular and renal injury. Oxid. Med. Cell. Longev..

[112] Tan S.M., de Haan J.B. (2014). Combating oxidative stress in diabetic complications with Nrf2 activators: How much is too much?. Redox Rep. Commun. Free Radic. Res..

[113] Nezu M., Suzuki N., Yamamoto M. (2017). Targeting the KEAP1-NRF2 system to prevent kidney disease progression. Am. J. Nephrol..

[114] Cui W., Min X., Xu X., Du B. (2017). Role of nuclear factor erythroid 2-related factor 2 in diabetic nephropathy. J. Diabetes Res..

[115] Zhou X., An G., Lu X. (2015). Hydrogen sulfide attenuates the development of diabetic cardiomyopathy. Clin. Sci..

[116] Xie L., Gu Y., Wen M., Zhao S., Wang W., Ma Y., Meng G., Han Y., Wang Y., Liu G. (2016). Hydrogen sulfide induces Keap1 S-sulfhydration and suppresses diabetes-accelerated atherosclerosis via Nrf2 activation. Diabetes.

[117] Yang H., Mao Y., Tan B., Luo S., Zhu Y. (2015). The protective effects of endogenous hydrogen sulfide modulator, S-propargyl-cysteine, on high glucose-induced apoptosis in cardiomyocytes: A novel mechanism mediated by the activation of Nrf2. Eur. J. Pharmacol..

[118] Cao X., Nie X., Xiong S., Cao L., Wu Z., Moore P.K., Bian J.S. (2018). Renal protective effect of polysulfide in cisplatin-induced nephrotoxicity. Redox Biol..

[119] Perez-Morales R.E., Del Pino M.D., Valdivielso J.M., Ortiz A., Mora-Fernandez C., Navarro-Gonzalez J.F. (2018). Inflammation in diabetic kidney disease. Nephron.

[120] Donath M.Y. (2016). Multiple benefits of targeting inflammation in the treatment of type 2 diabetes. Diabetologia.

[121] Pickup J.C., Crook M.A. (1998). Is type II diabetes mellitus a disease of the innate immune system?. Diabetologia.

[122] Alicic R.Z., Johnson E.J., Tuttle K.R. (2018). Inflammatory mechanisms as new biomarkers and therapeutic targets for diabetic kidney disease. Adv. Chronic Kidney Dis..

[123] Garcia-Garcia P.M., Getino-Melian M.A., Dominguez-Pimentel V., Navarro-Gonzalez J.F. (2014). Inflammation in diabetic kidney disease. World J. Diabetes.

[124] Navarro J.F., Mora C. (2005). Role of inflammation in diabetic complications. Nephrol. Dial. Transplant. Off. Publ. Eur. Dial. Transplant. Assoc. Eur. Ren. Assoc..

[125] Mora C., Navarro J.F. (2006). Inflammation and diabetic nephropathy. Curr. Diabetes Rep..

[126] Wang M., Tang W., Zhu Y.Z. (2017). An update on AMPK in hydrogen sulfide pharmacology. Front. Pharmacol..

[127] Sen U., Basu P., Abe O.A., Givvimani S., Tyagi N., Metreveli N., Shah K.S., Passmore J.C., Tyagi S.C. (2009). Hydrogen sulfide ameliorates hyperhomocysteinemia-associated chronic renal failure. Am. J. Physiol. Ren. Physiol..

[128] Toba H., Lindsey M.L. (2019). Extracellular matrix roles in cardiorenal fibrosis: Potential therapeutic targets for CVD and CKD in the elderly. Pharmacol. Ther..

[129] Hayashi T., Takai S., Yamashita C. (2010). Impact of the renin-angiotensin-aldosterone-system on cardiovascular and renal complications in diabetes mellitus. Curr. Vasc. Pharmacol..

[130] Kundu S., Pushpakumar S.B., Tyagi A., Coley D., Sen U. (2013). Hydrogen sulfide deficiency and diabetic renal remodeling: Role of matrix metalloproteinase-9. Am. J. Physiol. Endocrinol. Metab..

[131] Srivastava S.P., Koya D., Kanasaki K. (2013). MicroRNAs in kidney fibrosis and diabetic nephropathy: Roles on EMT and EndMT. BioMed Res. Int..

[132] Morgado-Pascual J.L., Marchant V., Rodrigues-Diez R., Dolade N., Suarez-Alvarez B., Kerr B., Valdivielso J.M., Ruiz-Ortega M., Rayego-Mateos S. (2018). Epigenetic modification mechanisms involved in inflammation and fibrosis in renal pathology. Mediators Inflamm.

[133] Kanasaki K., Shi S., Kanasaki M., He J., Nagai T., Nakamura Y., Ishigaki Y., Kitada M., Srivastava S.P., Koya D. (2014). Linagliptin-mediated DPP-4 inhibition ameliorates kidney fibrosis in streptozotocin-induced diabetic mice by inhibiting endothelial-to-mesenchymal transition in a therapeutic regimen. Diabetes.

[134] Harris R.C., Neilson E.G. (2006). Toward a unified theory of renal progression. Annu. Rev. Med..

[135] Zeisberg E.M., Potenta S.E., Sugimoto H., Zeisberg M., Kalluri R. (2008). Fibroblasts in kidney fibrosis emerge via endothelial-to-mesenchymal transition. J. Am. Soc. Nephrol. JASN.

[136] Villeneuve L.M., Reddy M.A., Natarajan R. (2011). Epigenetics: Deciphering its role in diabetes and its chronic complications. Clin. Exp. Pharmacol. Physiol..

[137] Kanasaki K., Nagai T., Nitta K., Kitada M., Koya D. (2014). N-acetyl-seryl-aspartyl-lysyl-proline: A valuable endogenous anti-fibrotic peptide for combating kidney fibrosis in diabetes. Front. Pharmacol..

[138] Li J., Qu X., Bertram J.F. (2009). Endothelial-myofibroblast transition contributes to the early development of diabetic renal interstitial fibrosis in streptozotocin-induced diabetic mice. Am. J. Pathol..

[139] Zhao L., Zhao J., Wang X., Chen Z., Peng K., Lu X., Meng L., Liu G., Guan G., Wang F. (2016). Serum response factor induces endothelial-mesenchymal transition in glomerular endothelial cells to aggravate proteinuria in diabetic nephropathy. Physiol. Genom..

[140] Zeisberg E.M., Tarnavski O., Zeisberg M., Dorfman A.L., McMullen J.R., Gustafsson E., Chandraker A., Yuan X., Pu W.T., Roberts A.B. (2007). Endothelial-to-mesenchymal transition contributes to cardiac fibrosis. Nat. Med..

[141] Li J., Bertram J.F. (2010). Review: Endothelial-myofibroblast transition, a new player in diabetic renal fibrosis. Nephrology.

[142] Medici D., Kalluri R. (2012). Endothelial-mesenchymal transition and its contribution to the emergence of stem cell phenotype. Semin. Cancer Biol..

[143] Ying R., Wang X.Q., Yang Y., Gu Z.J., Mai J.T., Qiu Q., Chen Y.X., Wang J.F. (2016). Hydrogen sulfide suppresses endoplasmic reticulum stress-induced endothelial-to-mesenchymal transition through Src pathway. Life Sci..

[144] Kalluri R., Weinberg R.A. (2009). The basics of epithelial-mesenchymal transition. J. Clin. Investig..

[145] Zeisberg M., Bottiglio C., Kumar N., Maeshima Y., Strutz F., Muller G.A., Kalluri R. (2003). Bone morphogenic protein-7 inhibits progression of chronic renal fibrosis associated with two genetic mouse models. Am. J. Physiol. Ren. Physiol..

[146] Yeung K.T., Yang J. (2017). Epithelial-mesenchymal transition in tumor metastasis. Mol. Oncol..

[147] Carew R.M., Wang B., Kantharidis P. (2012). The role of EMT in renal fibrosis. Cell Tissue Res..

[148] Loeffler I., Wolf G. (2015). Epithelial-to-mesenchymal transition in diabetic nephropathy: Fact or fiction?. Cells.

[149] Fragiadaki M., Mason R.M. (2011). Epithelial-mesenchymal transition in renal fibrosis—Evidence for and against. Int. J. Exp. Pathol..

[150] Zeisberg M., Hanai J., Sugimoto H., Mammoto T., Charytan D., Strutz F., Kalluri R. (2003). BMP-7 counteracts TGF-beta1-induced epithelial-to-mesenchymal transition and reverses chronic renal injury. Nat. Med..

[151] Hills C.E., Squires P.E. (2011). The role of TGF-beta and epithelial-to mesenchymal transition in diabetic nephropathy. Cytokine Growth Factor Rev..

[152] Lin S., Visram F., Liu W., Haig A., Jiang J., Mok A., Lian D., Wood M.E., Torregrossa R., Whiteman M. (2016). GYY4137, a slow-releasing hydrogen sulfide donor, ameliorates renal damage associated with chronic obstructive uropathy. J. Urol..

[153] Lin S., Lian D., Liu W., Haig A., Lobb I., Hijazi A., Razvi H., Burton J., Whiteman M., Sener A. (2018). Daily therapy with a slow-releasing H_2_S donor GYY4137 enables early functional recovery and ameliorates renal injury associated with urinary obstruction. Nitric Oxide Biol. Chem..

[154] Lin S., Juriasingani S., Sener A. (2018). Is hydrogen sulfide a potential novel therapy to prevent renal damage during ureteral obstruction?. Nitric Oxide Biol. Chem..

[155] Huang Y., Zhang Z., Huang Y., Mao Z., Yang X., Nakamura Y., Sawada N., Mitsui T., Takeda M., Yao J. (2018). Induction of inactive TGF-beta1 monomer formation by hydrogen sulfide contributes to its suppressive effects on Ang II- and TGF-beta1-induced EMT in renal tubular epithelial cells. Biochem. Biophys. Res. Commun..

[156] Guo L., Peng W., Tao J., Lan Z., Hei H., Tian L., Pan W., Wang L., Zhang X. (2016). Hydrogen sulfide inhibits transforming growth Factor-Beta1-induced EMT via Wnt/Catenin pathway. PLoS ONE.

[157] Lee H.J., Mariappan M.M., Feliers D., Cavaglieri R.C., Sataranatarajan K., Abboud H.E., Choudhury G.G., Kasinath B.S. (2012). Hydrogen sulfide inhibits high glucose-induced matrix protein synthesis by activating AMP-activated protein kinase in renal epithelial cells. J. Biol. Chem..

[158] Pfeilschifter J. (1995). Does nitric oxide, an inflammatory mediator of glomerular mesangial cells, have a role in diabetic nephropathy?. Kidney Int. Suppl..

[159] Ha H., Kim K.H. (1999). Pathogenesis of diabetic nephropathy: The role of oxidative stress and protein kinase C. Diabetes Res. Clin. Pract..

[160] Makino H., Sugiyama H., Kashihara N. (2000). Apoptosis and extracellular matrix-cell interactions in kidney disease. Kidney Int. Suppl..

[161] Sugiyama H., Kashihara N., Maeshima Y., Okamoto K., Kanao K., Sekikawa T., Makino H. (1998). Regulation of survival and death of mesangial cells by extracellular matrix. Kidney Int..

[162] Guan Y., Breyer M.D. (2001). Peroxisome proliferator-activated receptors (PPARs): Novel therapeutic targets in renal disease. Kidney Int..

[163] Kobori H., Nangaku M., Navar L.G., Nishiyama A. (2007). The intrarenal renin-angiotensin system: From physiology to the pathobiology of hypertension and kidney disease. Pharmacol. Rev..

[164] Malek V., Sharma N., Sankrityayan H., Gaikwad A.B. (2019). Concurrent neprilysin inhibition and renin-angiotensin system modulations prevented diabetic nephropathy. Life Sci..

[165] De Morais R.B., do Couto Muniz V.P., Nunes Costa E., Filho S.R.F., Nakamura Hiraki K.R., Bispo-da-Silva L.B., Coelho Balbi A.P. (2018). Mast cell population in the development of diabetic nephropathy: Effects of renin angiotensin system inhibition. Biomed. Pharmacother..

[166] Brewster U.C., Perazella M.A. (2004). The renin-angiotensin-aldosterone system and the kidney: Effects on kidney disease. Am. J. Med..

[167] Ohashi N., Urushihara M., Satou R., Kobori H. (2010). Glomerular angiotensinogen is induced in mesangial cells in diabetic rats via reactive oxygen species—ERK/JNK pathways. Hypertens. Res. Off. J. Jpn. Soc. Hypertens..

[168] Catherwood M.A., Powell L.A., Anderson P., McMaster D., Sharpe P.C., Trimble E.R. (2002). Glucose-induced oxidative stress in mesangial cells. Kidney Int..

[169] D’Araio E., Shaw N., Millward A., Demaine A., Whiteman M., Hodgkinson A. (2014). Hydrogen sulfide induces heme oxygenase-1 in human kidney cells. Acta Diabetol..

[170] Ding T., Chen W., Li J., Ding J., Mei X., Hu H. (2017). High glucose induces mouse mesangial cell overproliferation via inhibition of hydrogen sulfide synthesis in a TLR-4-dependent manner. Cell. Physiol. Biochem. Int. J. Exp. Cell. Physiol. Biochem. Pharmacol..

[171] Reddy G.R., Kotlyarevska K., Ransom R.F., Menon R.K. (2008). The podocyte and diabetes mellitus: Is the podocyte the key to the origins of diabetic nephropathy?. Curr. Opin. Nephrol. Hypertens..

[172] Wolf G., Chen S., Ziyadeh F.N. (2005). From the periphery of the glomerular capillary wall toward the center of disease: Podocyte injury comes of age in diabetic nephropathy. Diabetes.

[173] Jefferson J.A., Shankland S.J., Pichler R.H. (2008). Proteinuria in diabetic kidney disease: A mechanistic viewpoint. Kidney Int..

[174] Hishikawa A., Hayashi K., Itoh H. (2018). Transcription factors as therapeutic targets in chronic kidney disease. Molecules.

[175] Turkmen K. (2017). Inflammation, oxidative stress, apoptosis, and autophagy in diabetes mellitus and diabetic kidney disease: The four horsemen of the apocalypse. Int. Urol. Nephrol..

[176] Susztak K., Raff A.C., Schiffer M., Bottinger E.P. (2006). Glucose-induced reactive oxygen species cause apoptosis of podocytes and podocyte depletion at the onset of diabetic nephropathy. Diabetes.

[177] Stitt-Cavanagh E., MacLeod L., Kennedy C. (2009). The podocyte in diabetic kidney disease. Sci. World J..

[178] Chen Y., Liu C.P., Xu K.F., Mao X.D., Lu Y.B., Fang L., Yang J.W., Liu C. (2008). Effect of taurine-conjugated ursodeoxycholic acid on endoplasmic reticulum stress and apoptosis induced by advanced glycation end products in cultured mouse podocytes. Am. J. Nephrol..

[179] Inoki K., Mori H., Wang J., Suzuki T., Hong S., Yoshida S., Blattner S.M., Ikenoue T., Ruegg M.A., Hall M.N. (2011). mTORC1 activation in podocytes is a critical step in the development of diabetic nephropathy in mice. J. Clin. Investig..

[180] Hartleben B., Godel M., Meyer-Schwesinger C., Liu S., Ulrich T., Kobler S., Wiech T., Grahammer F., Arnold S.J., Lindenmeyer M.T. (2010). Autophagy influences glomerular disease susceptibility and maintains podocyte homeostasis in aging mice. J. Clin. Investig..

[181] Godel M., Hartleben B., Herbach N., Liu S., Zschiedrich S., Lu S., Debreczeni-Mor A., Lindenmeyer M.T., Rastaldi M.P., Hartleben G. (2011). Role of mTOR in podocyte function and diabetic nephropathy in humans and mice. J. Clin. Investig..

[182] Liu Y., Zhao H., Qiang Y., Qian G., Lu S., Chen J., Wang X., Guan Q., Liu Y., Fu Y. (2015). Effects of hydrogen sulfide on high glucose-induced glomerular podocyte injury in mice. Int. J. Clin. Exp. Pathol..

[183] Qian X., Li X., Ma F., Luo S., Ge R., Zhu Y. (2016). Novel hydrogen sulfide-releasing compound, S-propargyl-cysteine, prevents STZ-induced diabetic nephropathy. Biochem. Biophys. Res. Commun..

[184] Lee H.J., Feliers D., Mariappan M.M., Sataranatarajan K., Choudhury G.G., Gorin Y., Kasinath B.S. (2015). Tadalafil integrates nitric oxide-hydrogen sulfide signaling to inhibit high glucose-induced matrix protein synthesis in podocytes. J. Biol. Chem..

[185] Nasri H., Rafieian-Kopaei M. (2014). Metformin and diabetic kidney disease: A mini-review on recent findings. Iran. J. Pediatr..

[186] Wilinski B., Wilinski J., Somogyi E., Piotrowska J., Opoka W. (2013). Metformin raises hydrogen sulfide tissue concentrations in various mouse organs. Pharmacol. Rep. PR.

[187] John A., Kundu S., Pushpakumar S., Fordham M., Weber G., Mukhopadhyay M., Sen U. (2017). GYY4137, a hydrogen sulfide donor modulates miR194-dependent collagen realignment in diabetic kidney. Sci. Rep..

[188] Qian L.L., Liu X.Y., Chai Q., Wang R.X. (2018). Hydrogen sulfide in diabetic complications: Focus on molecular mechanisms. Endocr. Metab. Immune Disord. Drug Targets.

[189] Kanwar Y.S., Sun L., Xie P., Liu F.Y., Chen S. (2011). A glimpse of various pathogenetic mechanisms of diabetic nephropathy. Annu. Rev. Pathol..

[190] Sharma K. (2014). Obesity, oxidative stress, and fibrosis in chronic kidney disease. Kidney Int. Suppl..

[191] Dimas G.G., Didangelos T.P., Grekas D.M. (2017). Matrix gelatinases in atherosclerosis and diabetic nephropathy: Progress and challenges. Curr. Vasc. Pharmacol..

[192] Lloberas N., Cruzado J.M., Franquesa M., Herrero-Fresneda I., Torras J., Alperovich G., Rama I., Vidal A., Grinyo J.M. (2006). Mammalian target of rapamycin pathway blockade slows progression of diabetic kidney disease in rats. J. Am. Soc. Nephrol. JASN.

[193] Lee M.J., Feliers D., Mariappan M.M., Sataranatarajan K., Mahimainathan L., Musi N., Foretz M., Viollet B., Weinberg J.M., Choudhury G.G. (2007). A role for AMP-activated protein kinase in diabetes-induced renal hypertrophy. Am. J. Physiol. Ren. Physiol..

[194] Eid A.A., Ford B.M., Block K., Kasinath B.S., Gorin Y., Ghosh-Choudhury G., Barnes J.L., Abboud H.E. (2010). AMP-activated protein kinase (AMPK) negatively regulates Nox4-dependent activation of p53 and epithelial cell apoptosis in diabetes. J. Biol. Chem..

[195] Tain Y.L., Lee C.T., Chan J.Y., Hsu C.N. (2016). Maternal melatonin or N-acetylcysteine therapy regulates hydrogen sulfide-generating pathway and renal transcriptome to prevent prenatal N (G)-Nitro-L-arginine-methyl ester (L-NAME)-induced fetal programming of hypertension in adult male offspring. Am. J. Obstet. Gynecol..

